# Novel robust time series analysis for long-term and short-term prediction

**DOI:** 10.1038/s41598-021-91327-8

**Published:** 2021-06-07

**Authors:** Hiroshi Okamura, Yutaka Osada, Shota Nishijima, Shinto Eguchi

**Affiliations:** 1 Fisheries Resources Institute, Japan Fisheries Research and Education Agency, 2-12-4 Fukuura, Kanazawa, Yokohama, Kanagawa 236-8648 Japan; 2 The Institute of Statistical Mathematics, 10-3 Midori-cho, Tachikawa, Tokyo, 190-8562 Japan

**Keywords:** Computational biology and bioinformatics, Ecology, Ecology, Environmental sciences, Ocean sciences

## Abstract

Nonlinear phenomena are universal in ecology. However, their inference and prediction are generally difficult because of autocorrelation and outliers. A traditional least squares method for parameter estimation is capable of improving short-term prediction by estimating autocorrelation, whereas it has weakness to outliers and consequently worse long-term prediction. In contrast, a traditional robust regression approach, such as the least absolute deviations method, alleviates the influence of outliers and has potentially better long-term prediction, whereas it makes accurately estimating autocorrelation difficult and possibly leads to worse short-term prediction. We propose a new robust regression approach that estimates autocorrelation accurately and reduces the influence of outliers. We then compare the new method with the conventional least squares and least absolute deviations methods by using simulated data and real ecological data. Simulations and analysis of real data demonstrate that the new method generally has better long-term and short-term prediction ability for nonlinear estimation problems using spawner–recruitment data. The new method provides nearly unbiased autocorrelation even for highly contaminated simulated data with extreme outliers, whereas other methods fail to estimate autocorrelation accurately.

## Introduction

Nonlinear modeling is widely applied to ecological data analysis. The spawner-recruitment (SR) relationship, which is also called the stock-recruitment relationship, is fundamental to population dynamics modeling, risk assessments in conservation biology, and sustainable use of wildlife^1–3^. A nonlinear curve is frequently used to model SR relationships. However, it is notoriously difficult to estimate SR relationships accurately, especially for many fish stocks^4,5^, given that SR data often have large deviations around the SR curve, including extreme outliers, and residuals left over after SR curve fitting are likely to show strong autocorrelation. These outliers and autocorrelations make statistical inference difficult and rarely permit accurate estimation of existing SR relationships hidden in the data of a single population.

Meta-analysis of information from multiple independent studies^6^ is an influential method for overcoming the difficulty of finding evidence of density dependence from individual population data. In contrast with individual analysis, meta-analysis of SR data elicits firm conclusions and facilitates evidence-based decision making by integrating the parameters and/or results obtained by fitting a nonlinear model to time series data on abundance and SR relationships^7,8^. For instance, Brook & Bradshaw (2006) fitted nonlinear models to the abundance time series data of 1198 species and integrated the results to detect evidence for density dependence^9^. Although they found that density dependence is a pervasive feature of population dynamics in various species, support for the density dependence of fish was generally weaker than those of other taxa (the average relative support for density dependence was 74.7%, whereas that of fish was 60.1%, which was the least among all taxa). This lack of power to detect density dependence could be caused by excessive noise (i.e., large observation and process errors), outliers, and the complex correlation structure involved in individual SR relationships in fish. The improvement in quality of the result from an individual dataset using a robust estimation method against noisy data is therefore of crucial importance even in meta-analysis.

Predictive ability based on SR relationships is closely related to sustainable fisheries management. Setting proper long-term management objectives is necessary for sustainable use of fish, whereas fishers and fish processors are usually concerned with short-term prediction of catches for logistics and planning for the next season. Balancing the trade-offs between long-term and short-term management objectives will therefore enhance compliance with fisheries regulations, thereby avoiding overfishing and consequently realizing sustainability goals. The credibility and benefits of long-term management objectives depend on the accuracy and precision of the parameter estimates of SR relationships, which provide biological reference points based on maximum sustainable yield. In contrast, short-term predictive ability is strongly influenced by the magnitude and autocorrelation of residuals as well as parameter estimates of the SR relationship. Achieving accurate and precise long-term and short-term prediction from noisy SR data contributes substantially to the sustainable use of fish. A robust method previously developed for estimating fish recruitment^10^ cannot handle autocorrelated residuals, and subsequent research demonstrated that the method was highly sensitive to small perturbations in the SR data^11^. A regression model that is insensitive to perturbations that deviate from the mean relationship and is sensitive to the autocorrelation of residuals must mitigate the trade-offs between long-term and short-term objectives for sustainable development.

**Figure 1:**
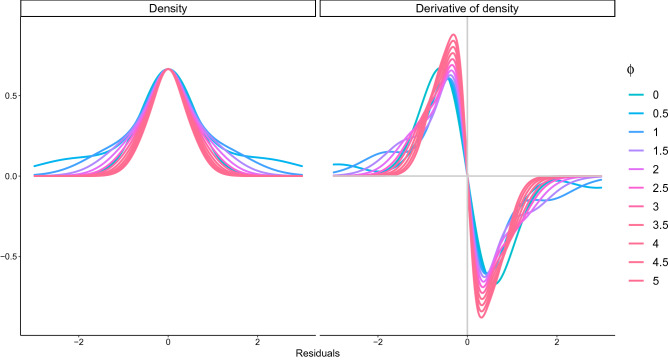
The probability density function and its derivatives for various $\phi$ parameters.

**Figure 2:**
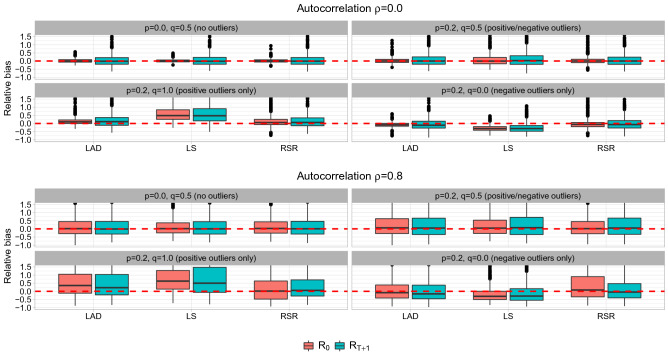
Relative bias of $R_0$ (the quantity related to the long-term conservation objective) and $R_{T+1}$ (the quantity related to the short-term conservation objective) for the simulation using the HS SR function with/without autocorrelation and outliers in the residuals.

In this work, we propose a new robust regression approach by extending a least squares (LS) method to realize a weighted maximum likelihood method with changeable variance and autocorrelation dependent on deviance residuals. The new approach accurately estimates parameters and precisely predicts autocorrelated error structures, even for contaminated SR relationship data containing many outliers. The approach is tested using simulated SR data having some outliers and autocorrelated error structures. The results are compared with those from traditional regression models that use an LS method and a least absolute deviations (LAD) method. Further, we apply our robust regression approach to compiled fish spawner-recruitment data from Japan^12^.

## Methods

The data needed for estimating the SR relationship consist of spawning biomass (*S*) and recruitment (*R*) observed over time. A lognormal distribution is frequently used as the distribution of errors for SR relationships^13^. We therefore assume that the residuals from a regression model having $r=\log (R)$ as a response variable and the logarithm of the latent SR relationship as the mean will have a normal distribution. In addition, we assume that the latent SR relationship is likely to be contaminated by some outliers given that fish populations often suffer from nonnegligible contamination, such as sporadic strong cohorts^5^.

**Figure 3:**
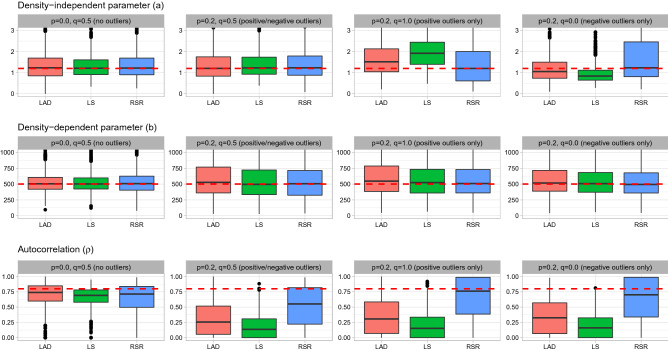
Parameter estimates of the density-independent parameter (*a*), density-dependent parameter (*b*), and autocorrelation ($\rho$) for the simulation using the HS SR function with autocorrelation (true $\rho = 0.8$) in the residuals.

**Figure 4:**
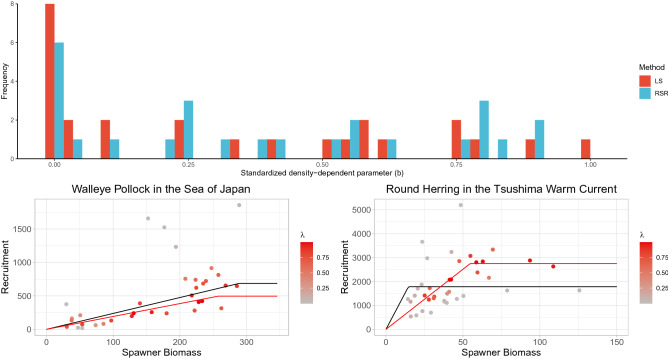
Application of the robust SR model to fish population data from Japan. (Top) Estimates of $(b-\min (S))/(\max (S)-\min (S))$ using the LS and RSR methods. (Bottom) Examples of fitted SR curves using the LS (black line) and RSR (red line) methods (left, walleye pollock in the Sea of Japan; right, round herring in the Tsushima warm current).

### A robust regression approach

Suppose that the logarithm of recruitment ($r_t = \log (R_t), \ (t = 1, \ldots , T)$) has the following autocorrelated normal distribution,

1 $$\begin{aligned} r_t = f(S_t|{\varvec{\theta }})+\varepsilon _t, \end{aligned}$$

where $\varepsilon _t$ is a scaled autoregressive error of order one, that is, $\sqrt{\lambda _t}(\varepsilon _t-\rho \sqrt{\lambda _{t-1}} \varepsilon _{t-1})= e_t$ with a gaussian noise $e_t$ of mean zero and variance $\sigma ^2$, $S_t$ is the spawning biomass, $f(S_t|{\varvec{\theta }})$ is the logarithm of a density-dependent population growth model (spawner-recruitment (SR) curve), ${\varvec{\theta }}$ is the parameter (vector) of the SR curve, $\rho$ is the autocorrelation, and $\sigma ^2$ is the base variance of the normal distribution. $\lambda _t \, (\in (0,1])$ is the weight for a datum in year *t*. Rearranging the equation for $\varepsilon _t$, we have $\varepsilon _t \sim N(\rho \sqrt{\lambda _{t-1}} \varepsilon _{t-1}, \sigma ^2/\lambda _t)$ (Appendix A). We define $\lambda _t$ to be related to the magnitude of the residual $\varepsilon _t$,

$$\begin{aligned} \lambda _t = \exp \left( - \phi \varepsilon _t^2 \right) , \end{aligned}$$

where $\phi \, (>0)$ is the parameter that adjusts the influence of outliers. Given that the base variance $\sigma ^2$ is divided by $\lambda _t$, the variance is inflated when the difference between the datum and the SR curve is large. The model is equivalent to the AR(1) model when $\lambda _t \equiv 1$ (i.e., $\phi =0$) for any *t*. $\sqrt{\lambda _t}$ is interpreted as the probability of the datum being generated from an uncontaminated normal distribution. When changing the $\phi$ parameter with $\rho =0$, the shapes of the probability density function and its derivative are similar to the Tukey’s biweight (also called bisquare) function^14^, which is close to the gaussian function near zero but decays swiftly as the datum becomes farther from zero (Fig. 1).

By solving the equation at equilibrium, the mean deviance residual at $t=1$ is zero and the variance at $t=1$ is given by ${\text{var}}({\varepsilon_{1}} ){ = }{\sigma ^{2}} {{/}}\left[ {\lambda _{1}} \left( {1} - {\rho ^{2}} {\tilde{\lambda }} \right) \right]$, where ${\tilde{\lambda }}$ is calculated by substituting the sample mean of $\lambda _t$, $\tilde{\lambda } = (1/T) \sum _{t=1}^T \lambda _t$ (Appendix B). Incorporating the initial status, the log-likelihood function to be maximized is given by

2 $$\begin{aligned} \log (L) = \sum _{t=1}^T \log \left( N(r_t|f(S_t|{\varvec{\theta }})+\delta _t, \nu _t \sigma ^2 \lambda _t^{-1}) \right) , \end{aligned}$$

where $\delta _{t} = 0$ and $\nu _{t} = (1-\rho ^2 \tilde{\lambda })^{-1}$ if $t = 1$, and $\delta _{t} = \rho \sqrt{\lambda _{t-1}} \varepsilon _{t-1}$ and $\nu _{t} = 1$ if $t > 1$. Because $\varepsilon _{t-1}$ increases and $\lambda _{t-1}$ decreases when there is an outlier at $t-1$, the multiplication of $\rho$ and $\sqrt{\lambda _{t-1}}$ mitigates the influence of an extreme outlier on autocorrelation and contributes to the restoration of the original autocorrelation.

We need to estimate the parameters $\sigma$, $\rho$, and $\phi$ in addition to the SR relationship parameters ${\varvec{\theta }}$. The parameter $\phi$ determines the mixing proportion of contamination and governs the predictive ability of the model. We use time series cross-validation^15^, which is also called retrospective forecasting^16^ (RF), to stably determine the value of $\phi$. First we delete the last datum. Then we use the SR relationship estimated from the data excluding the last datum to forecast recruitment and calculate its error assuming that the deleted recruitment for the last year is true. Next, we delete the two last data, forecast the second-to-last recruitment, and calculate the error assuming that the deleted second-to-last year’s recruitment is true. After the procedure is repeated on a rolling basis, the $\phi$ parameter having the smallest average error is finally selected. The optimum $\phi$ is determined by minimizing the following RF error:

3 $$\begin{aligned} RF_R = \exp \left( \frac{1}{P} \sum _{t=1}^P \log \left[ \left( r_{T-(t-1)} -\hat{r}_{T-(t-1)}^{1:(T-t)} \right) ^2 \right] \right) . \end{aligned}$$

This is the geometric mean of predicted errors, which stabilizes the performance of retrospective forecasting. $r_{T-(t-1)}$ is the logarithm of observed recruitment in year $T-(t-1)$ and $\hat{r}_{T-(t-1)}^{1:(T-t)}$ is the predicted value estimated using the data from years 1 to $T-t$, which is given by

$$\begin{aligned} \hat{r}_{T-(t-1)}^{1:(T-t)} = f(S_{T-(t-1)}|\hat{\varvec{\theta }})+\hat{\rho } \sqrt{\hat{\lambda }_{T-t}} \hat{\varepsilon }_{T-t}, \end{aligned}$$

where $t = 1, \ldots , P$. We adopt $P=10$ for stable estimation in this paper, though we commonly take 5 as the minimum *P*^17^.

All subsequent analyses are performed using R^18^ and its package TMB^19^ (Template Model Builder).

### Simulation

We generate the simulated data ($\left\{ (R_t, S_t) ; t = 1, \ldots , T \right\}$) with some outliers and autocorrelated errors and test the performance of our robust SR (RSR) method in comparison with the LS and LAD methods. LAD was chosen because it is a typical robust method and is generally superior to the least median squares method used in Chen & Paloheimo (1995)^11^. The average recruitment data are generated from the Hockey–Stick (HS) SR function^12^, $f(S_t|{\varvec{\theta }}) = \log \left( a \min (S_t, b) \right)$, where ${\varvec{\theta }} = (a, b) = (1.2, 500)$. Stochastic normal errors are added to the log recruitment data with or without autocorrelation. When there is an autocorrelation in the residuals of log recruitment, the autocorrelation is set to $\rho = 0.8$. To examine the effect of outliers, we add the outliers that occur at the expected frequency of twice per 10 years ($p=0.2$) to the residuals of log recruitment. The patterns of outlier occurrence are threefold: evenly occurring positive and negative outliers ($q=0.5$), all positive outliers ($q=1.0$), and all negative outliers ($q=0.0$) (see Appendix C for the definition of *q*). We then have eight types of simulated data (no outliers, positive and negative outliers, all positive outliers, and all negative outliers for autocorrelation in the normal residual $\rho = 0$ and $\rho =0.8$, respectively). The simulations are replicated 1,000 times for each of the eight types. The length of each SR data time series (*T*) is set to 30 years which is typical for SR time series data^9,12^. The performance of the methods is evaluated by two indicators that represent long-term and short-term predictive abilities $(\hat{R}_0 - R_0)/R_0$ and $(\hat{R}_{T+1} - R_{T+1})/R_{T+1}$, respectively, where the former is the asymptotic maximum recruitment ($R_0 = ab$ for the HS SR function) and the latter is recruitment in the ensuing year $T+1$, which is given by $R_{T+1} = \exp (f(S_{T+1}|{\varvec{\theta }}) + \rho \omega _{T} + \eta _{T+1})$, where $\omega _T$ and $\eta _{T+1}$ are independent gaussian noises (Appendix C). Note that the true recruitment at $T+1$ does not include any outliers. The mathematical details of the simulation are given in Appendix C. Autocorrelation is always estimated such that $\rho$ is set to zero when an estimate of $\rho$ is equal to or less than zero because a negative autocorrelation is usually impractical^20^. The parameter $\log (\phi )$ in RSR is chosen from the grid values from $-3.0$ to 3.0 in increments of 0.5. The best $\phi$ is a minimizer of the RF error $RF_R$ (Eq. 3).

For sensitivity tests, we conduct the following additional simulations: (S1) same as the above base case scenario (S0) except that $a = 1.8$; (S2) same as S0 except that $p = 0.1$ (the expected frequency of outliers is once every 10 years) in place of $p=0.2$; (S3) same as S0 except that $p = 0.3$ (the expected frequency of outliers is three times every 10 years) in place of $p=0.2$; (S4) same as S0 except that $f(S_t|{\varvec{\theta }})$ is the logarithm of the Beverton–Holt function; (S5) same as S0 except that $f(S_t|{\varvec{\theta }})$ is the logarithm of the Ricker function; S6) same as S0 except for the spawner-abundance dependent *p*, in which the expected frequency of outliers is higher for lower spawner abundances than for higher spawner abundances.

Finally, we calculate biological reference points related to maximum sustainable yield (MSY), i.e., fishing rate at MSY ($F_{\rm {msy}}$) and spawning biomass at MSY ($S_{\rm {msy}}$), for each scenario and evaluate their relative biases. To calculate $F_{\rm {msy}}$ and $S_{\rm {msy}}$, we require additional information on survival and growth as well as an assumption about population dynamics. For simplicity, we use the delay-difference model as the population dynamics model^5^. The mathematical details are given in Appendix D.

### Real data analysis

Ichinokawa, Okamura & Kurota (2017) fitted the SR curves to fish population data from Japan which comprise 26 SR datasets (Appendix E), demonstrating that some populations showed strong density dependence but others had weak or low density dependence. We fit the HS SR curves to the same 26 SR datasets used in Ichinokawa, Okamura & Kurota (2017). Because Ichinokawa, Okamura & Kurota (2017) used LS as the fitting method, we use LS and RSR to compare the density-independent parameter $\log (\hat{a})$, standardized density-dependent parameter $( \hat{b}-\min (S) )/( \max (S) - \min (S) )$, autocorrelation in the residuals $\hat{\rho }$, and predictability $\hat{RF}_R$ in the HS SR curves.

## Results

### Simulation

When the simulated data are generated without autocorrelation and outliers in the residuals, LS performs best because the true and estimation models are then entirely in agreement, and LAD and RSR also produce nearly unbiased results with only slightly worse precision (Fig. 2). When there are positive and negative outliers in the residuals but no autocorrelation, LAD, LS, and RSR still give nearly unbiased estimates for $R_0$ and $R_{T+1}$, but the precision of LS worsens in comparison with LAD and RSR. When the outliers are one-sided (positive alone or negative alone), LS shows biased results for both $R_0$ and $R_{T+1}$, whereas LAD and RSR still produce nearly unbiased estimates. As a whole, LAD and RSR show very similar results for simulated data without autocorrelation.

When the simulated data are generated with autocorrelated residuals and no outliers/two-sided outliers, LS, LAD, and RSR produce nearly unbiased estimates with similar accuracy and precision (Fig. 2). LAD provides biased results for the scenario with positive outliers alone. LS shows the best performance for scenarios with no outliers and balanced outliers but provides biased results for the scenarios with positive or negative outliers alone, similar to the results without autocorrelation. RSR provides nearly unbiased results for all scenarios and shows the best overall performance, although the precision of $R_0$ estimates for the scenario with negative outliers alone shows a small amount of deterioration.

When there is high autocorrelation ($\rho =0.8$) in the residuals, the density-dependent parameter *b* is estimated with almost no bias for all estimation methods and all scenarios, even though the precision of the LAD method is inferior to other methods (Fig. 3). All estimation methods provide nearly unbiased estimates about density-independent parameter *a* for the scenarios with no outliers and balanced outliers. However, the LS method shows biased estimates for the *a* parameter in the scenarios with positive or negative outliers alone and the LAD and RSR methods provide nearly unbiased *a* estimates, except that the LAD method produces slightly biased and less precise estimates for the scenario with positive outliers alone, whereas the RSR method produces slightly biased and less precise estimates for the scenario with negative outliers alone. The estimated $\rho$ parameters show a striking contrast among the three estimation methods. Although the LAD and LS methods provide autocorrelation estimates close to the true value for the scenario without outliers, they produce substantial underestimation of autocorrelation for the scenarios with outliers. In contrast, the RSR method produces nearly unbiased autocorrelation estimates for all scenarios, indicating that it results in good performance of the $R_{T+1}$ estimation (Fig. 2). When the outliers are two-sided, the autocorrelation tends to be underestimated even when using RSR, which is likely because the distribution of outliers is symmetrical, making it difficult to differentiate between normal errors and outliers. When there is no autocorrelation ($\rho =0.0$) or even moderate autocorrelation ($\rho =0.4$), the general tendency of the results is invariant such that LS is sensitive to outliers and only the RSR can estimate autocorrelation accurately (Appendix F).

Sensitivity analyses show qualitatively consistent results similar to the base case scenario (Appendix G). LAD shows good performance on unbiased estimation of the long-term prediction but generally has worse performance on the short-term prediction and less precision for both $R_0$ and $R_{T+1}$, particularly when there is autocorrelation. LS provides good performance on long-term and short-term prediction when there are no one-sided outliers but produces biased estimates when there are one-sided outliers. RSR shows nearly unbiased estimates for both long-term and short-term predictions and is generally the best performer. When there are both autocorrelation and outliers in the dataset simultaneously, only the RSR is able to estimate the autocorrelation accurately. When the density-independent parameter *a* is increased, the results hardly change. When the expected frequency is once every 10 years ($p=0.1$), the precision ameliorates for all methods, but the general trends are invariant except for the performance of LAD, which greatly improves even when there is autocorrelation. When the expected frequency is three times every 10 years ($p=0.3$), the precision deteriorates for all methods but the general trends are invariant except for the performance of LAD, which slightly worsens when there is autocorrelation. In contrast, RSR is insensitive to the change of *p*. When the SR function is Beverton-Holt or Ricker, the general results are similar to those of the base case except that the precision worsens when the SR function is Beverton-Holt and there is autocorrelation. When the expected frequency of outliers is higher for lower spawner abundances than for higher spawner abundances^4^, the general trends are still similar, although the accuracy and the precision become slightly worse. Again, RSR is insensitive to this change.

Because biological reference points such as $F_{\rm {msy}}$ and $S_{\rm {msy}}$ are closely related to the SR parameters *a* and *b* (Appendix D), LAD and LS having biased SR parameter estimates (Fig. 3) overestimate $F_{\rm {msy}}$ and underestimate $S_{\rm {msy}}$ when there are positive outliers, and vice versa when there are negative outliers (Appendix H). RSR generally produces nearly unbiased $F_{\rm {msy}}$ and $S_{\rm {msy}}$ estimates. Although the degree of bias is generally smaller compared with SR curve parameters (Fig. 3) probably due to the effects of other parameters, the bias of LS in particular is large for scenarios with large uncertainties (S3 and S6).

### Real data analysis

The average values of the density-independent parameters $\log (a)$ for 26 populations are 1.566 (SD: 2.510) for LS and 1.539 (SD: 2.444) for RSR. The average values of the density-dependent parameters $(b-\min (S))/(\max (S)-\min (S))$ are 0.330 (SD: 0.335) for LS and 0.392 (SD: 0.328) for RSR. The average values of the autocorrelation $\rho$ are 0.482 (SD: 0.316) for LS and 0.433 (SD: 0.411) for RSR. The average values of retrospective forecasting bias $RF_R$ are 0.246 (SD: 0.189) for LS and 0.189 (SD: 0.136) for RSR. Thus, RSR decreased the frequency of extreme density-dependent parameter estimates relative to LS (Fig. 4) and improved the predictability in terms of retrospective forecasting. Although the overall change is not so large, the impact of using RSR on individual populations can be great. For example, whereas the LS-based SR curve for walleye pollock (*Gadus chalcogrammus*) in the Sea of Japan shows a linear relationship (no density-dependence), the RSR-based SR curve shows a break point within the observed spawner abundances (Fig. 4). In contrast, although the LS-based SR curve for round herring (*Etrumeus teres*) in the Tsushima warm current shows a flat relationship (extremely strong density-dependence), the RSR-based SR curve also shows a break point within the observed spawner abundances (Fig. 4).

The parameters *a* and *b* are closely related to $F_{\rm{{msy}}}$ and $S_{\rm{{msy}}}$ (Appendix D). The estimated *a* and *b* for walleye pollock are $\hat{a}=2.38$ and $(\hat{b}-\min (S))/(\max (S)-\min (S))=1.00$ for LS and $\hat{a}=1.93$ and $(\hat{b}-\min (S))/(\max (S)-\min (S))=0.88$ for RSR, whereas those for round herring are $\hat{a}=122.2$ and $(\hat{b}-\min (S))/(\max (S)-\min (S))=0.003$ for LS and $\hat{a}=50.2$ and $(\hat{b}-\min (S))/(\max (S)-\min (S))=0.364$ for RSR. This suggests that $F_{\rm{{msy}}}$ would be lower for both species and $S_{\rm{{msy}}}$ would be lower for walleye pollock and higher for round herring if RSR is used for SR curve fitting, indicating a substantial change in management objectives.

## Discussion

The RSR produces nearly unbiased estimates with the SR and autocorrelation parameters and shows the best performance in terms of long-term and short-term predictions for the simulated data with autocorrelation and outliers in comparison with the LS and LAD methods. Because the SR data are usually autocorrelated and generally have many outliers^5^, an RSR that is robust to outliers and can accurately estimate autocorrelation in the residuals would be a welcome development in ecology. This robustness and unbiasedness are caused by the new residual error structure for overdispersion and the new handling of autocorrelation (Eq. 2). The expected performance of an RSR for the long-term prediction on $R_0$ and the short-term prediction on $R_{T+1}$ permits us to overcome the trade-offs between long-term and short-term ecological objectives. Although LAD generally shows good performance for base SR parameter estimation, particularly when the occurrence frequency of outliers is 10% (Appendix F), using the RSR instead of LAD even for such cases is advantageous because the RSR can predict the probability of outlier occurrence through the $\lambda$ parameter and would be useful in future predictions for strategic fish management and conservation planning.

Both LAD and LS overestimate $F_{\rm{{msy}}}$ and underestimate $S_{\rm{{msy}}}$ when there are positive outliers, while RSR provides unbiased $F_{\rm{{msy}}}$ and $S_{\rm{{msy}}}$ estimates (Appendix H). This means that LAD and LS would tend to overfish the stock beyond MSY if $F_{\rm{{msy}}}$ and/or $S_{\rm{{msy}}}$ are used as management targets. However, when there are negative outliers, LS and LAD produce the opposite biases, and would thus produce more conservative management targets, which could benefit population sustainability while sacrificing annual available harvest. Additionally, RSR appears to produce more variable estimates than the other methods under some scenarios (Appendix H), and thus sometimes produces biased estimates for individual simulations, even though the median estimate is unbiased. This indicates that RSR may produce biased estimates of long-term management targets in some situations, even if it performs better on average and produces better short-term management targets. Thus, it is desirable to develop a method to further stabilize the estimates of RSR in the future.

The approach used in this paper is applicable to various robust regression problems, not to mention a linear regression with outliers. Although we dealt with just a one-year time lag or autoregressive process of order 1 in this paper, the RSR method is easily extended to an autoregressive process with higher orders AR(*p*) using $\sum _{i=1}^{p} \rho _i \sqrt{\lambda _{t-i}} \varepsilon _{t-i}$ in Eqs. 1 and 2. Loss functions other than least squares and a normal distribution are frequently used for robust regression methods^14,21,22^. Although we used a normal distribution in this case, we can use other probabilistic distributions in our modeling framework. The time series cross-validation or retrospective forecasting for selecting the optimal $\phi$ parameter worked well for our simulation trials and analyses of real data. The efficient factor used for selecting the threshold parameter of robust regression in Wang et al. (2018) might also be used in our method. Comparisons between our RSR method and traditional robust M regression methods such as those of Tukey and Huber^14^ will be topics of future research.

A state-space model (SSM) is frequently used to model nonlinear population dynamics^23–27^. Although SSMs are very attractive and useful even for robust regressions^23^, differentiating observation and process errors using only a single time series is notoriously difficult, particularly for nonlinear modeling^5^. Because the length of a time series for estimating an SR curve is usually short and is 100 years at most, RSR is advantageous for single species analyses. Application of RSR to real data led to a change in estimated density-dependent parameters (Fig. 4) and, as a result, the management targets for sustainable use and conservation will also change when the biological reference points are used. Given that meta-analysis is a synthesis of multiple independent studies, using a robust regression method such as RSR for individual studies contributes to better inference and prediction for meta-analysis using a global database. However, integrating information from multiple data sources by hierarchical modeling can lead to different perspectives compared with the aggregation of independent outputs^27^. Because RSR can be incorporated into the SSM framework, integration of RSR and SSM would therefore be a promising approach to realize more stable and accurate analyses.

Robust nonlinear regression analysis is potentially applicable to extensive ecological time series data, including not only SR data as in the present work, but also radioisotope contamination data^28^. Ecological data are often simultaneously contaminated by inevitable but obstructive outliers and influenced by autocorrelative phenomena. The outliers make long-term and short-term prediction difficult, whereas autocorrelation affects the long-term and short-term prediction and may even distort the estimation results of the latent nonlinear structure. Traditional robust regression approaches alleviate the influence of outliers but make estimation of autocorrelation difficult. Our RSR reduces the influence of outliers and accurately estimates the innate autocorrelation, thereby greatly improving long-term and short-term prediction ability compared with traditional robust regression approaches. Accordingly, RSR holds promise for extensive applications and may prove useful for various ecological problems.

## Supplementary Information

Supplementary Information.
