# Supplementary material for: Novel robust time series analysis for long-term and short-term prediction
Source: Sci Rep. 2021 Jun 7;11:11938. doi: 10.1038/s41598-021-91327-8 (PMC8184922; doi:10.1038/s41598-021-91327-8)
Supplement: Supplementary file 1 — Supplementary Information. [file 41598_2021_91327_MOESM1_ESM.pdf]

Novel Robust Time Series Analysis for Long-term and Short-term Prediction

Hiroshi Okamura   Yutaka Osada   Shota Nishijima
Shinto Eguchi

Appendix A: A numerical simulation for generating random numbers from the error distribution in Equation 1

Random numbers from the error distribution in Eq. 1,

$$\varepsilon_t \sim N(\rho\sqrt{\lambda_{t-1}}\varepsilon_{t-1}, \sigma^2/\lambda_t),$$

are generated from the following simple sampling–importance–resampling (SIR) algorithm.

[A simple SIR algorithm for generating random numbers from the error distribution in Eq. 1]

1. Suppose that we have $\varepsilon_{t-1,k}$ and $\lambda_{t-1,k}$ $(k = 1, \ldots, n)$.
2. Generate random number $u_{i,k}$ $(i = 1, ..., M)$ from a uniform distribution, $u_{i,k} \sim \text{Unif}[-B, B]$.
3. Then calculate the weight $\lambda_{i,k} = \exp(-\phi u_{i,k}^2)$.
4. Next calculate the probability density $N(u_{i,k}|\rho\sqrt{\lambda_{t-1,k}}\varepsilon_{t-1,k}, \sigma^2/\lambda_{i,k})$.
5. Generate $\varepsilon_{t,k}$ $(k = 1, \ldots n)$ randomly using the above probability densities as the weight distribution.

We ran the above algorithm with $n = 1000$, $M = 100,000$, $B = 4.0$, $\varepsilon_{0,k} = 0.0$ $(\forall k)$, $\rho = 0.8$, and $\sigma = 0.4$. The length of each time series was set to $T = 100$. The means and covariances of generated time series for different $\phi = (0.0, 0.1, 0.5, 1.0, 1.5, 2.0, 2.5, 3.0, 3.5)$ after eliminating the initial 50 years as burn-in samples show approximate weak (or second-order) stationarity where the covariance $\text{cov}(\varepsilon_{t+l}, \varepsilon_t)$ was calculated for $l = 0, 1, \ldots, 5$ (Figures A1 and A2). The lag $l = 0$ corresponds to the variance, $\text{cov}(\varepsilon_t, \varepsilon_t) = \text{var}(\varepsilon_t)$. Note that the error distribution is equivalent to the AR(1) process when $\phi = 0.0$ and the variance at equilibrium is $\sigma^2/(1 - \rho^2) = 0.444$.

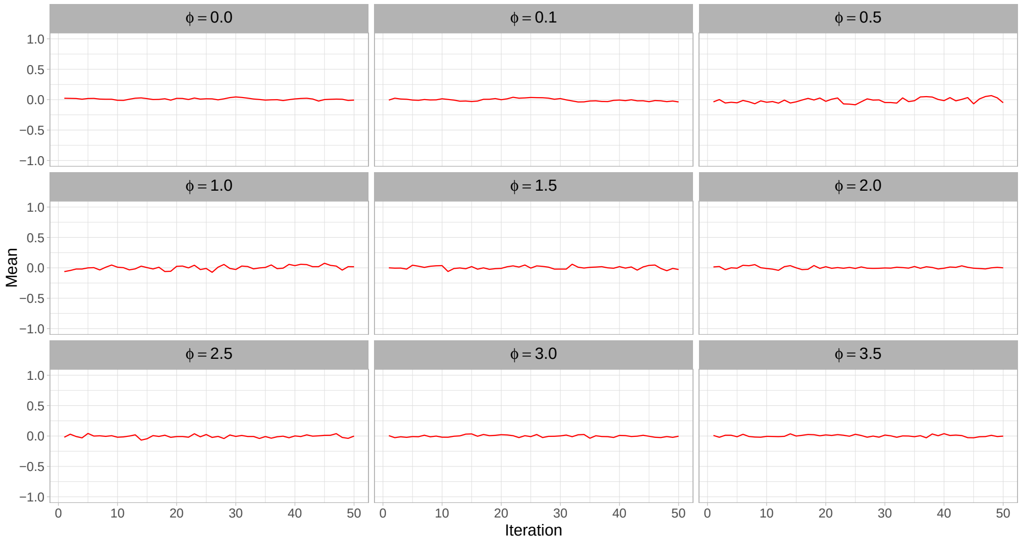

Figure A1: The mean of time series for iterations of the simulation ($t = 51, \dots, 100$) for $\phi = (0.0, 0.1, 0.5, 1.0, 1.5, 2.0, 2.5, 3.0, 3.5)$.

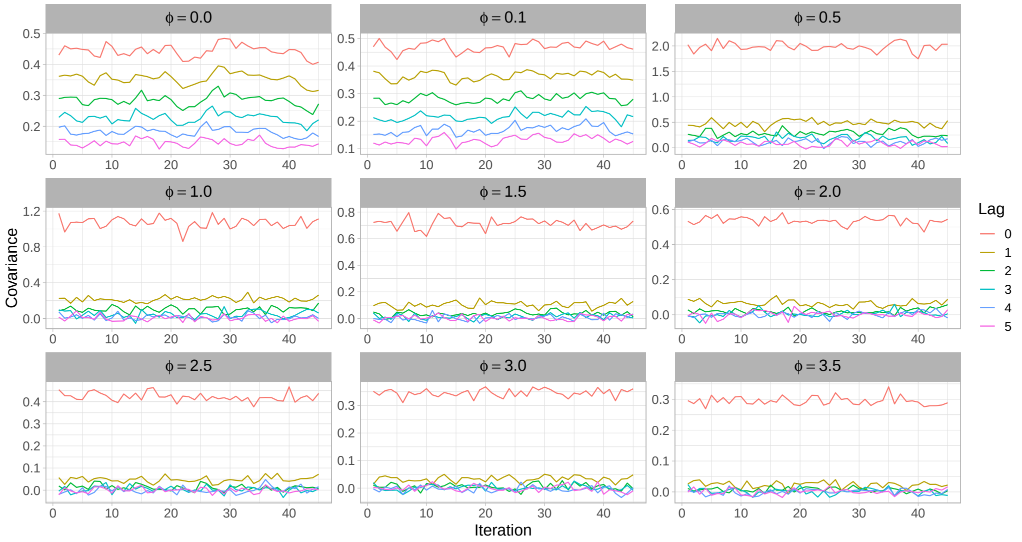

Figure A2: The covariance (lag $= 0, \dots, 5$) of time series for iterations of the simulation ($t = 51, \dots, 95$) for $\phi = (0.0, 0.1, 0.5, 1.0, 1.5, 2.0, 2.5, 3.0, 3.5)$.

Appendix B: Derivation of variance at equilibrium

Residuals obtained by multiplying Eq. 1 by $\sqrt{\lambda_t}$ are given by

$$\sqrt{\lambda_t}\varepsilon_t = \rho\sqrt{\lambda_t}\sqrt{\lambda_{t-1}}\varepsilon_{t-1} + e_t,$$

where $e_t \sim N(0, \sigma^2)$. Taking the variance for the equilibrium at $t = 1$ and approximating λ_t to be constant λ,

$$\mathrm{var}(\sqrt{\lambda}\varepsilon) = \rho^2\lambda\mathrm{var}(\sqrt{\lambda}\varepsilon) + \sigma^2.$$

Solving the equation for $\mathrm{var}(\sqrt{\lambda}\varepsilon)$,

$$\mathrm{var}(\sqrt{\lambda}\varepsilon) = \frac{\sigma^2}{(1-\rho^2\lambda)}.$$

Because λ on the right-hand side of the above equation is unobserved, we substitute the sample mean of λ_t, $\tilde{\lambda} = (1/T)\sum_{t=1}^{T}\lambda_t = (1/T)\sum_{t=1}^{T}\exp(-\phi\varepsilon_t^2)$. (Here, although we can use statistics other than the sample mean, the simulation results were robust to other statistics we examined). We then obtain

$$\mathrm{var}(\varepsilon_1) = \frac{\sigma^2}{\lambda_1(1-\rho^2\tilde{\lambda})}.$$

When using the random number generation in Appendix A, we do not see any serious deviations from the comparison between the true probability $N(\varepsilon_t|\rho\sqrt{\lambda_{t-1}}\varepsilon_{t-1}, \sigma^2/\lambda_t)$ and the approximate probability $N(\varepsilon_t|0, \sigma^2/(\lambda_1(1-\rho^2\tilde{\lambda})))$ at the time $t = 51$ (Figure A3).

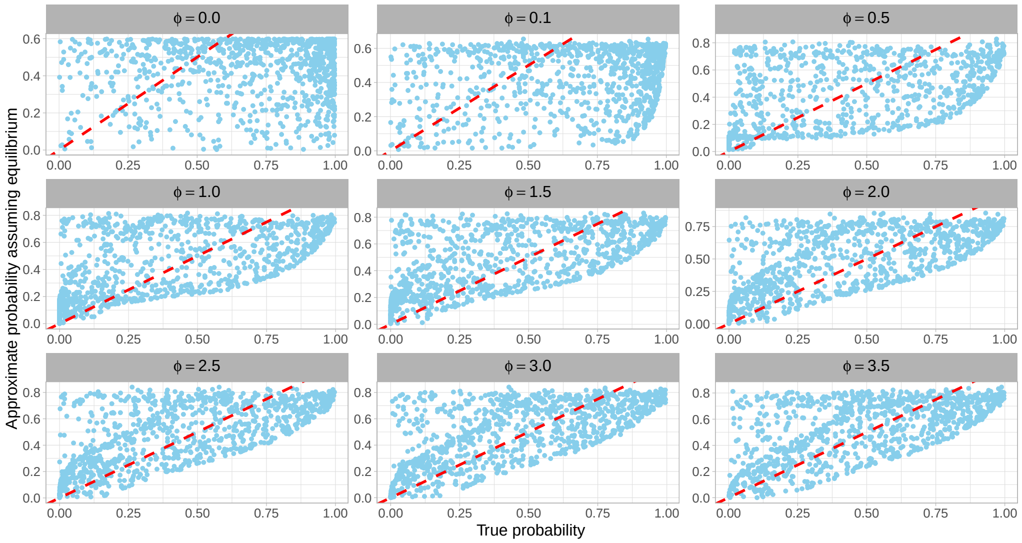

Figure A3: Comparison between the true probability $N(\varepsilon_t|\rho\sqrt{\lambda_{t-1}}\varepsilon_{t-1}, \sigma^2/\lambda_t)$ and the approximate probability $N(\varepsilon_t|0, \sigma^2/(\lambda_1(1-\rho^2\tilde{\lambda})))$. The broken red line is $y = x$

Appendix C: The simulation specification

Spawner–recruitment (SR) data having some outliers and latently autocorrelated residuals were generated in the following way. First, we generated the spawner data from a multivariate normal distribution.

$$\log(S_t) \sim MVN(A + Bt + d_t, \omega^2\Sigma),$$

where $A = \log(1500)$, $B = -0.1$, $d_t \sim N(0, 0.4^2)$, $\omega = 0.4^2$, and

$$\Sigma = \begin{pmatrix} 1 & & \xi^{\Delta t} \\ & \ddots & \\ \xi^{\Delta t} & & 1 \end{pmatrix},$$

where ξ takes a value between 0 and 1 and is the correlation between adjacent spawners. Δt is the difference in year t. (Thus, the correlation of one year difference is ξ, the correlation of two years difference is ξ^2, and so on.) ξ was set to 0.8.

Using the generated spawner time series, the recruitment time series were generated by

$$R_t = \exp\left(f(S_t|\boldsymbol{\theta}) + \varepsilon_t\right),$$

where ε_t is the annual variation between recrutiments. The errors ε_t include an autocorrelation and outliers as follows:

$$\varepsilon_t = \begin{cases} \omega_t & (z_t = 0) \\ \psi_t & (z_t = 1) \end{cases},$$

where ω_t represents a normal but autocorrelated error and is modeled by

$$\omega_t = \rho\omega_{t-1} + \eta_t, \quad \eta_t \sim N(0, \sigma^2).$$

ψ_t represents an abnormal outlier and is modelled by

$$\psi_t = (2u_t - 1)\zeta_t, \quad \zeta_t \sim N(\mu, \tau^2),$$

where ζ_t is contamination with the mean μ, where μ was set to 2 in our simulation. z_t is a random variable that takes 0 or 1 and controls the probability of outlier occurrence, and u_t is also a binary random variable that controls the sign of the outliers. Both z_t and u_t have a binomial distribution:

$$z_t \sim Bin(1, p),$$

$$u_t \sim Bin(1, q),$$

where p is the probability that an outlier occurs and q determines an occurrence ratio of positive and negative outliers ($q = 0.5$, an equally expected number of positive and negative outliers; $q = 0.0$, all negative outliers; $q = 1.0$, all positive outliers).

When the spawner abundance is low, the recruitment variability tends to be higher (Myers 2001). To test it, the year-dependent p is modeled by

$$p_t = 2\alpha / \left[1 + \exp(-\beta(S_t - E(S_t))/SD(S_t))\right],$$

where $E(S_t)$ is the mean spawner abundance and $SD(S_t)$ is the standard deviation of spawner abundance time series. α is the mean frequency of outlier occurrence and β is a parameter that controls the dependence of outlier occurrence on the level of spawner abundance. When β is negative, outliers occur more frequently with a lower spawner abundance. β was set to zero ($p_t \equiv \alpha$, no dependence on spawner abundance) or -3 (greater occurrence of outliers when the spawner abundance is low).

We used the Hockey-Stick, Beverton-Holt, and Ricker functions (Ichinokawa, Okamura & Kurota 2017) for the SR relationship $f(S_t|\boldsymbol{\theta})$. For the Hockey-Stick function,

$$f(S_t|\boldsymbol{\theta}) = \log\left(a \min(S_t, b)\right);$$

for the Beverton-Holt function,

$$f(S_t|\boldsymbol{\theta}) = \log\left(aS_t/(1 + S_t/b)\right);$$

and for the Ricker function,

$$f(S_t|\boldsymbol{\theta}) = \log\left(aS_t \exp(-S_t/b)\right).$$

The SR parameter $\boldsymbol{\theta} = (a, b)$ where a is the density-independent parameter and b is the density-dependent parameter. b was set to 500 irrespective of the forms of SR function, whereas a was basically set to 1.2 but changed to 1.8 for one scenario in the simulation trials to evaluate its sensitivity.

The performance of the predictive models was evaluated by two indicators

$$\frac{\hat{R}_0 - R_0}{R_0},$$

and

$$\frac{\hat{R}_{T+1} - R_{T+1}}{R_{T+1}},$$

where the former is the asymptotic maximum recruitment ($R_0 = ab$ for the Hockey-Stick SR function and the Beverton-Holt SR function; and $R_0 = ab\exp(-1)$ for the Ricker SR function) and the latter is the recruitment in the ensuing year $T + 1$, which is given by $R_{T+1} = \exp(f(S_{T+1}|\boldsymbol{\theta}) + \rho\omega_T + \eta_{T+1})$, where $\eta_{T+1} \sim N(0, \sigma^2)$.

The simulation was conducted under the following six scenarios:

S0) $f(S_t|\boldsymbol{\theta})$ is the Hockey-Stick function. $a = 1.2$ and $b = 500$. $p = 0.2$ (= the expected frequency of outlier occurrence is twice every 10 years). The autocorrelation ρ is set to zero or 0.8.

S1) Same as S0 except that $a = 1.8$.

S2) Same as S0 except that $p = 0.1$ (the expected frequency of outliers is once every 10 years) in place of $p = 0.2$.

S3) Same as S0 except that $p = 0.3$ (the expected frequency of outliers is three times every 10 years) in place of $p = 0.2$.

S4) Same as S0 except that $f(S_t|\boldsymbol{\theta})$ is the logarithm of the Beverton-Holt function.

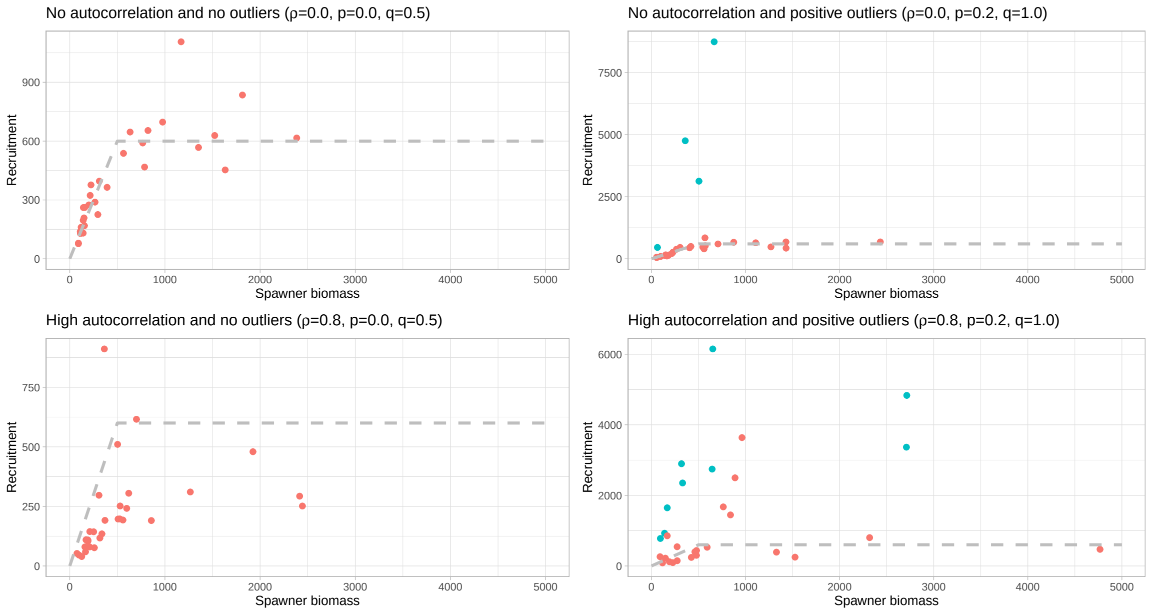

Figure A4: Examples of SR data for the basecase scenario (S0). The red dots are from the base SR curve with/without autocorrelated errors and the blue dots are from the contaminated SR curve with positive outliers. The broken gray line is the base HS SR curve.

S5) Same as S0 except that $f(S_t|\boldsymbol{\theta})$ is the logarithm of the Ricker function.

S6) Same as S0 except for $\beta = -3.0$ in p_t, where the expected frequency of outliers is higher for lower spawner abundances than for higher spawner abundances.

The patterns of $p = 0.0$ and $q = 0.5$ (no outliers), $p = 0.2$ and $q = 0.5$ (positive and negative outliers), $p = 0.2$ and $q = 1.0$ (positive outliers), and $p = 0.2$ and $q = 0.0$ (negative outliers) combined with the autocorrelation of residuals $\rho = 0.0$ or $\rho = 0.8$ for each scenario were investigated. (In total, eight patterns were tried for each scenario. $p = 0.2$ was replaced by $p = 0.1$ and $p = 0.3$ for Scenarios 2 and 3, respectively.) The simulation was replicated 1,000 times for each pattern in each scenario.

We fit three estimation methods (LS, LAD, and RSR) to the simulated data. When LS is applied, we use $\sqrt{1-\rho^2}\varepsilon_1 \sim N(0, \sigma^2)$ for $t = 1$ and $\varepsilon_t \sim N(\rho\varepsilon_{t-1}, \sigma^2)$ for $t > 1$, where N is the probability density function of the normal (Gaussian) distribution, $\varepsilon_t = r_t - f(S_t|\boldsymbol{\theta})$, ρ is the autocorrelation, and σ^2 is the variance. When LAD is applied, we use $\sqrt{1-\rho^2}\varepsilon_1 \sim L(0, \tau)$ for $t = 1$ and $\varepsilon_t \sim L(\rho\varepsilon_{t-1}, \tau)$ for $t > 1$, where L is the probability density function of the Laplace distribution, $\varepsilon_t = r_t - f(S_t|\boldsymbol{\theta})$, ρ is the autocorrelation, and τ is the scale parameter (Dielman & Rose 1994). When RSR is applied, we use the method described in the text. The parameters are estimated using a maximum likelihood approach for all methods.

The examples of SR data for the basecase scenario (S0) are shown in Figure A4, where the HS SR function was used.

Appendix D: Estimation of biological reference points

Calculation of biological reference points needs additional information related to the population dynamics. For simplicity, we use the general delay-difference model (Walters & Martell 2004) as the population dynamics model:

$$\begin{aligned} B_{t+1} &= s(\nu N_t + \phi B_t)(1 - F_t) + w_r R_{t+1}, \\ N_{t+1} &= s N_t (1 - F_t) + R_{t+1} \end{aligned}$$

where B_t is the biomass at year t, N_t is the abundance (number) at year t, s is the survival rate, and F_t is the fishing rate at year t. The growth parameters ν and ϕ are derived from the linear relationship for weght at age a, w_a, (a Ford-Walford plot):

$$w_a = \nu + \phi w_{a-1}$$

and w_r is the mean weight at recruitment age (r). We assume that $w_{r-1} = 0$ and $w_r = \nu$. The recruitment $R_t = f(S_{t-1})$, where the spawning biomass $S_t = B_t(1–F_t)$, is given by the HS curve ($f(S_{t-1}) = a \min(S_{t-1}, b)$), the Beverton-Holt curve ($f(S_{t-1}) = aS_{t-1}/(1 + S_{t-1}/b)$), or the Ricker curve ($f(S_{t-1}) = aS_{t-1} \exp(-S_{t-1}/b)$).

By eliminating the subscript t from the above equation and rearrainging the equation, the total biomass and spawning biomass at equilibrium are obtained. When the HS curve is used, depending on the biological parameters, the spawning biomass at maximum sustainable yield (S_{msy}) corresponds to the break point of the curve or S fished at $F_{\max}$ ($F_{\max}$ is the fishing rate that gives the maximum yield per recruit). When there is no growth effect, S_{msy} is always the break point. We demonstrate the mathematical derivation below.

The HS curve has a stable non-zero equilibrium when $S \geq b$, whereas it does not have a stable non-zero equilibrium when $S < b$. Thus, we first assume $S \geq b$ and then $R = ab$. The total biomass and the spawning biomass at equilibrium for the HS curve are

$$\tilde{B} = \frac{\nu ab}{\left[1 - s(1 - \tilde{F})\right]\left[1 - \phi s(1 - \tilde{F})\right]},$$

$$\tilde{S} = \frac{\nu ab(1 - \tilde{F})}{\left[1 - s(1 - \tilde{F})\right]\left[1 - \phi s(1 - \tilde{F})\right]}.$$

The sustainable yield is then

$$SY = \tilde{F}\tilde{B} = \frac{\nu ab\tilde{F}}{\left[1 - s(1 - \tilde{F})\right]\left[1 - \phi s(1 - \tilde{F})\right]}.$$

Thus, the maximum sustainable yield satisfies

$$\frac{d(SY)}{d\tilde{F}} = \frac{\nu ab\left[(1 - s)(1 - \phi s) - \phi s^2 \tilde{F}^2\right]}{\left\{\left[1 - s(1 - \tilde{F})\right]\left[1 - \phi s(1 - \tilde{F})\right]\right\}^2} = 0.$$

The solution of this equation is

$$\tilde{F} = \frac{1}{s}\sqrt{\frac{(1-s)(1-\phi s)}{\phi}}.$$

This fishing rate corresponds to $F_{\rm max}$ of the yield per recruitment (YPR: Quinn & Deriso 1999) curve because R is assumed to be constant ($R = ab$).

However, since HS has no stable non-zero equilibrium when $S < b$, $\tilde{S} \geq b$ needs to be satisfied for having a sustainable solution. So then, the following inequality is needed:

$$\nu a(1-\tilde{F}) \geq \left[1 - s(1-\tilde{F})\right]\left[1 - \phi s(1-\tilde{F})\right].$$

Rearranging this equation, we have

$$\phi s^2(1-\tilde{F})^2 - (s + \phi s + \nu a)(1-\tilde{F}) + 1 \leq 0.$$

Solving the equation leads to

$$0 \leq \tilde{F} \leq \max\left(1 - \frac{B - \sqrt{B^2 - 4AC}}{2A}, 0\right)$$

where $A = \phi s^2$, $B = s + \phi s + \nu a$, and $C = 1$. The upper boundary corresponds to the fishing rate that leads to $\tilde{S} = b$, referred to as $F_{\rm threshold}$ afterwards. To keep $\tilde{F} > 0$, $\nu a > (1-s)(1-\phi s)$. For any $\tilde{F}$, $\tilde{B} \geq 0$ such that $1 - \phi s > 0$ and then $\phi < 1/s$.

Thus, if $\frac{1}{s}\sqrt{\frac{(1-s)(1-\phi s)}{\phi}} > 1 - \frac{B-\sqrt{B^2-4AC}}{2A}$, $\tilde{F} = 1 - \frac{B-\sqrt{B^2-4AC}}{2A} = F_{\rm threshold}$ and if $\frac{1}{s}\sqrt{\frac{(1-s)(1-\phi s)}{\phi}} \leq 1 - \frac{B-\sqrt{B^2-4AC}}{2A}$, $\tilde{F} = \frac{1}{s}\sqrt{\frac{(1-s)(1-\phi s)}{\phi}} = F_{\rm max}$. Which one $F_{\rm msy}$ becomes is determined depending on the parameters νa,s, and ϕ. Generally $F_{\rm msy} = F_{\rm threshold}$ unless s and ϕ are high. When $\phi = 0$, the above inequality becomes

$$0 \leq \tilde{F} \leq \max\left(1 - 1/(s + \nu a), 0\right)$$

and $F_{\rm max} \to \infty$. Thus, since $d(SY)/d\tilde{F} > 0$, $F_{\rm msy} = \max\left(1 - 1/(s+\nu a), 0\right) = F_{\rm threshold}$. This means that the $S_{\rm msy}$ for the HS curve is always the break point if there is no growth effect.

Similarly, for the Beverton-Holt curve, the biomass and the spawning biomass at equilibrium are

$$\tilde{B} = b\left(\frac{\nu a}{\left[1 - s(1-\tilde{F})\right]\left[1 - \phi s(1-\tilde{F})\right]} - \frac{1}{1-\tilde{F}}\right),$$

$$\tilde{S} = b(1-\tilde{F})\left(\frac{\nu a}{\left[1 - s(1-\tilde{F})\right]\left[1 - \phi s(1-\tilde{F})\right]} - \frac{1}{1-\tilde{F}}\right).$$

The maximum sustainable yield then satisfies

$$\frac{d(SY)}{d\tilde{F}} = b\left(\frac{\nu a\left[(1-s)(1-\phi s) - \phi s^2\tilde{F}^2\right]}{\left\{\left[1 - s(1-\tilde{F})\right]\left[1 - \phi s(1-\tilde{F})\right]\right\}^2} - \frac{1}{(1-\tilde{F})^2}\right) = 0.$$

For the Ricker curve, the biomass and the spawning biomass at equilibrium are

$$\tilde{B} = \frac{b}{1-\tilde{F}} \log\left(\frac{\nu a(1-\tilde{F})}{\left[1-s(1-\tilde{F})\right]\left[1-\phi s(1-\tilde{F})\right]}\right),$$

$$\tilde{S} = b\log\left(\frac{\nu a(1-\tilde{F})}{\left[1-s(1-\tilde{F})\right]\left[1-\phi s(1-\tilde{F})\right]}\right).$$

When $G(\tilde{F}) = \log\left(\frac{\nu a(1-\tilde{F})}{\left[1-s(1-\tilde{F})\right]\left[1-\phi s(1-\tilde{F})\right]}\right)$, the maximum sustainable yield then satisfies

$$\frac{d(SY)}{d\tilde{F}} = b\left(\frac{G(\tilde{F})}{(1-\tilde{F})^2} - \frac{\tilde{F}\left(1-\phi s^2(1-\tilde{F})^2\right)}{\left[1-s(1-\tilde{F})\right]\left[1-\phi s(1-\tilde{F})\right](1-\tilde{F})^2}\right) = 0.$$

We use a numerical optimization to find the solution (F_{msy}) for the Beverton-Holt curve and the Ricker curve.

Here we assume $\nu = 1$ without loss of generality. We then set $s = 0.67$ and $\phi = 0.5$. We calculate F_{msy} and S_{msy}/S_0 where S_0 is the spawning biomass without fishing. The true $F_{\text{msy}} = 0.523$ and $S_{\text{msy}} = 0.183$ for the HS curve, $F_{\text{msy}} = 0.250$ and $S_{\text{msy}} = 0.317$ for the Beverton-Holt curve, and $F_{\text{msy}} = 0.316$ and $S_{\text{msy}} = 0.216$ for the Ricker curve when $a = 1.2$. The true $F_{\text{msy}} = 0.633$ and $S_{\text{msy}} = 0.122$ for the HS curve when $a = 1.8$ (Scenario S1). The results of estimated F_{msy} and S_{msy} for scenarios are given in Appendix H.

Appendix E: Real data

We fit the HS SR curves to the SR datasets of the 26 fish populations used in Ichinokawa, Okamura, & Kurota (2017). The population assessments were conducted by virtual population analysis without assuming any parametric SR relationship (Ichinokawa, Okamura, & Kurota 2017, Okamura, Yamashita, & Ichinokawa 2017).

Table A1: Population datasets ($N = 26$) used for the analysis of real data.

Stock ID	Common name	Scientific name	Region	Period
PILCHPJPN	Japanese sardine	Sardinops melanostictus	Pacific	1976–2013
PILCHTSST	Japanese sardine	Sardinops melanostictus	Tsushima Warm Current	1960–2013
JMACKPJPN	Japanese jack mackerel	Trachurus japonicus	Pacific	1982–2013
JMACKTSST	Japanese jack mackerel	Trachurus japonicus	Tsushima Warm Current	1973–2013
CMACKPJPN	Chub mackerel	Scomber japonicus	Pacific	1970–2013
CMACKTSST	Chub mackerel	Scomber japonicus	Tsushima Warm Current	1973–2013
SMACKPJPN	Spotted mackerel	Scomber australasicus	Pacific	1995–2013
SMACKECS	Spotted mackerel	Scomber australasicus	Tsushima Warm Current	1992–2013
APOLLPSOJ	Walleye pollock	Gadus chalcogramma	Sea of Japan	1980–2013
APOLLPJPN	Walleye pollock	Gadus chalcogramma	Pacific	1981–2013
RHERRTSST	Round herring	Etrumeus teres	Tsushima Warm Current	1976–2013
JANCHOPJPN	Japanese anchovy	Engraulis japonicus	Pacific	1978–2013
JANCHOSETO	Japanese anchovy	Engraulis japonicus	Seto Inland Sea	1981–2013
AMBERJ	Amberjack	Seriola quinqueradiata	Around Japan	1994–2013
RBRMSETOE	Red seabream	Pagrus major	Seto Inland Sea (east)	1977–2013
RBRMSETOW	Red seabream	Pagrus major	Seto Inland Sea (west)	1977–2013
RBRMECS	Red seabream	Pagrus major	Sea of Japan and East China Sea	1986–2013
SPANMACKSETO	Japanese Spanish mackerel	Scomberomorus niphonius	Seto Inland Sea	1987–2013
JFLOUNSETO	Japanese flounder	Paralichthys olivaceus	Seto Inland Sea	1994–2013
JFLOUNNSJ	Japanese flounder	Paralichthys olivaceus	Sea of Japan (north)	1999–2013
JFLOUNECS	Japanese flounder	Paralichthys olivaceus	Sea of Japan and East China Sea	1986–2013
RONOFLOUNSOJ	Round-nose flounder	Eopsetta grigorjewi	Sea of Japan	1993–2013
POFLOUNSOJ	Point-head flounder	Cleisthenes herzensteini	Sea of Japan	1997–2013
WILLFLOUNNP	Willowy flounder	Tanakius kitaharae	Pacific	1998–2013
JPPUFFSOJ	Japanese pufferfish	Takifugu rubripes	Sea of Japan/East China Sea/Seto Inland Sea	2002–2013
JPPUFFISE	Japanese pufferfish	Takifugu rubripes	Ise and Mikawa Bay	1993–2013

Appendix F: Results for different autocorrelations in the simulation

We show only the results for the autocorrelation in the simulation $\rho = 0.8$ in the main text (Fig. 3). Here, we show the results of parameter estimation for no autocorrelation ($\rho = 0.0$) and moderate autocorrelation ($\rho = 0.4$) using the HS SR function, the Beverton-Holt SR function, and the Ricker SR function, respectively.

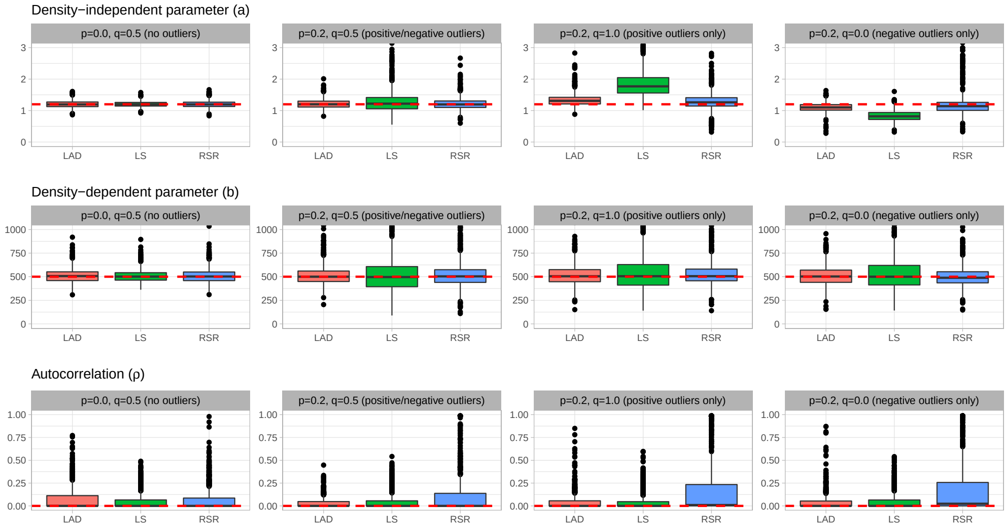

Figure A5: Parameter estimates of the density-independent parameter (a), density-dependent parameter (b), and autocorrelation (ρ) for the simulation using the HS SR function with autocorrelation (true $\rho = 0.0$) in the residuals.

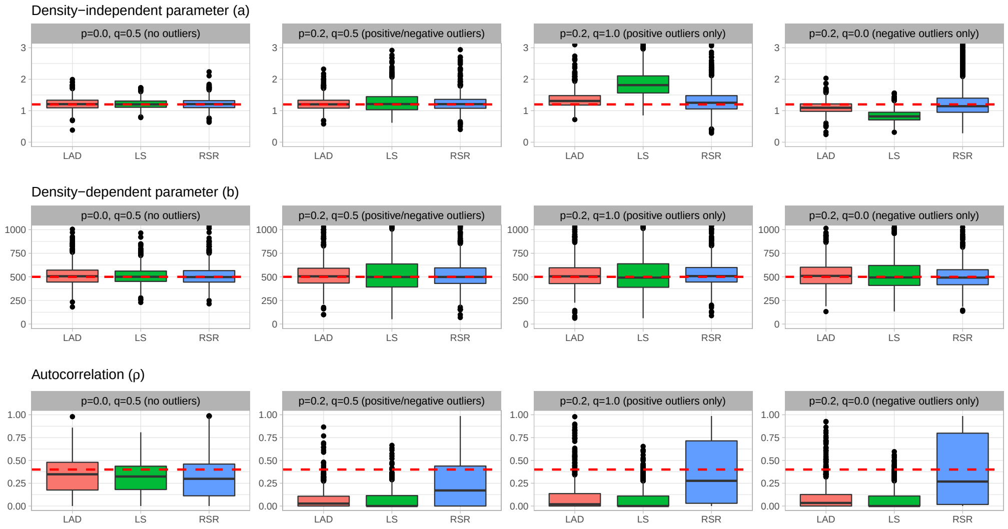

Figure A6: Parameter estimates of the density-independent parameter (a), density-dependent parameter (b), and autocorrelation (ρ) for the simulation using the HS SR function with autocorrelation (true $\rho = 0.4$) in the residuals.

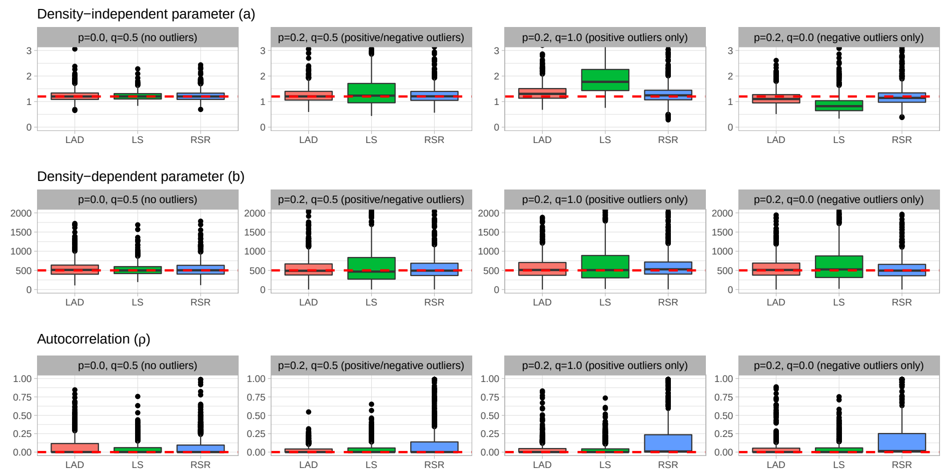

Figure A7: Parameter estimates of the density-independent parameter (a), density-dependent parameter (b), and autocorrelation (ρ) for the simulation using the Beverton-Holt SR function with autocorrelation (true $\rho = 0.0$) in the residuals.

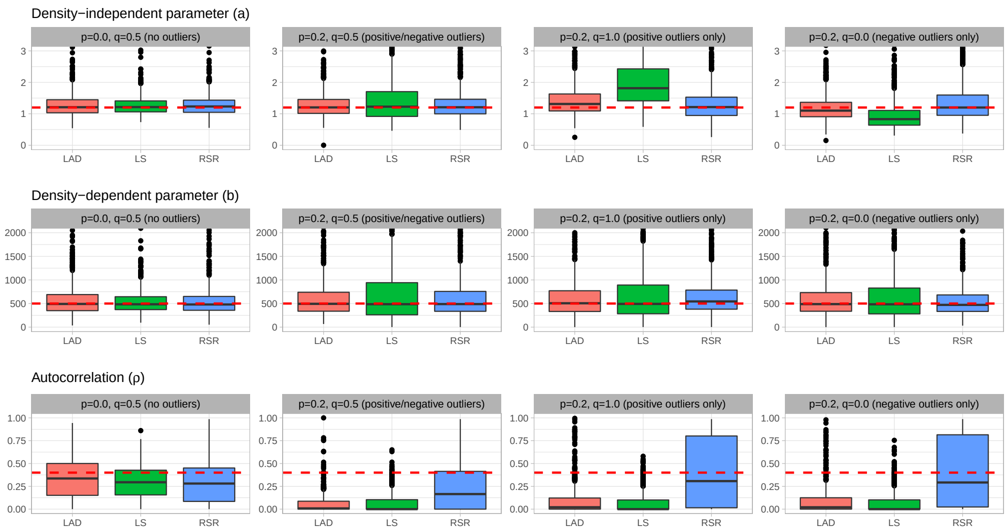

Figure A8: Parameter estimates of the density-independent parameter (a), density-dependent parameter (b), and autocorrelation (ρ) for the simulation using the Beverton-Holt SR function with autocorrelation (true $\rho = 0.4$) in the residuals.

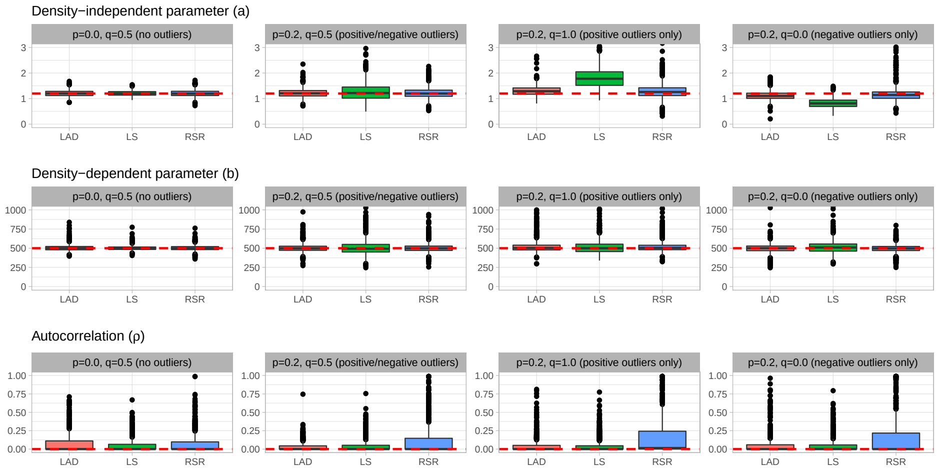

Figure A9: Parameter estimates of the density-independent parameter (a), density-dependent parameter (b), and autocorrelation (ρ) for the simulation using the Ricker SR function with autocorrelation (true $\rho = 0.0$) in the residuals.

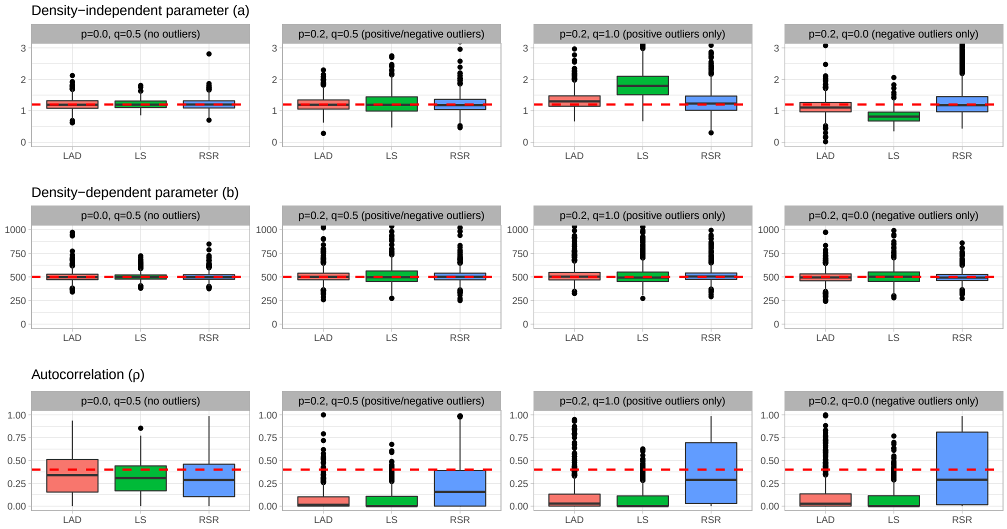

Figure A10: Parameter estimates of the density-independent parameter (a), density-dependent parameter (b), and autocorrelation (ρ) for the simulation using the Ricker SR function with autocorrelation (true $\rho = 0.4$) in the residuals.

Appendix G: Results of additional simulation tests

We show the performance statistics (relative bias of R_0 and R_{T+1}) and the estimated basic parameters (density-independent parameter a, density-dependent parameter b, and autocorrelation ρ for scenarios with autocorrelation in the residuals) for the sensitivity test scenarios (S1 – S6).

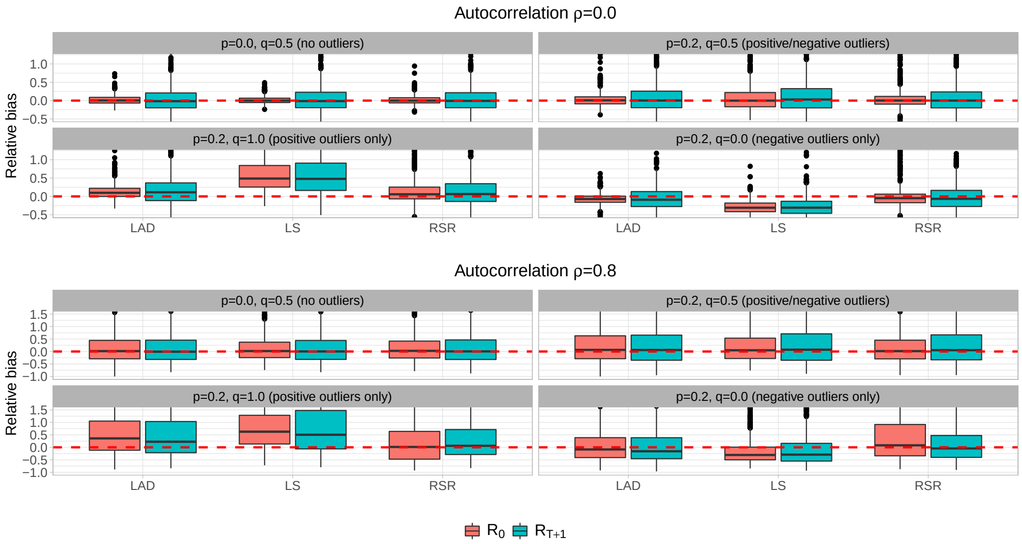

Figure A11: Relative bias of R_0 (the quantity related to the long-term conservation objective) and R_{T+1} (the quantity related to the short-term conservation objective) for the simulation using the HS SR function with/without autocorrelation and outliers in the residuals (S1: $a = 1.8$).

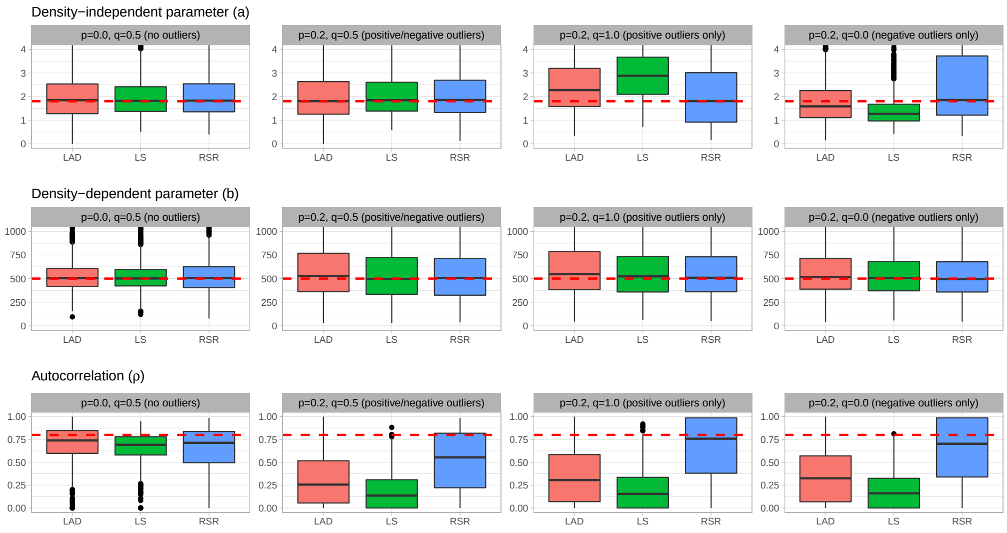

Figure A12: Parameter estimates of the density-independent parameter (a), density-dependent parameter (b), and autocorrelation (ρ) for the simulation using the HS SR function with autocorrelation (true $\rho = 0.8$) in the residuals (S1: $a = 1.8$).

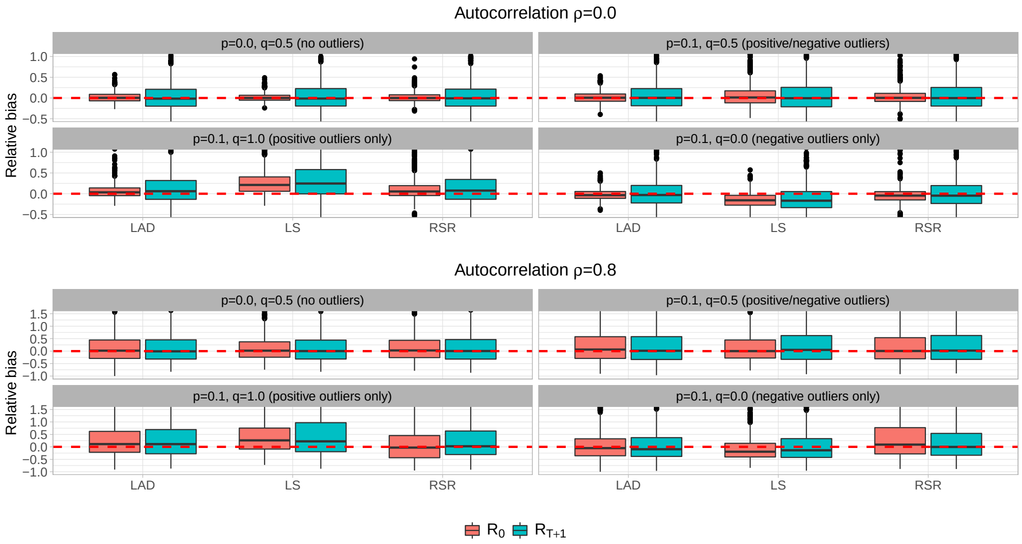

Figure A13: Relative bias of R_0 and R_{T+1} for the simulation using the HS SR function with/without autocorrelation and outliers in the residuals (S2: $p = 0.1$).

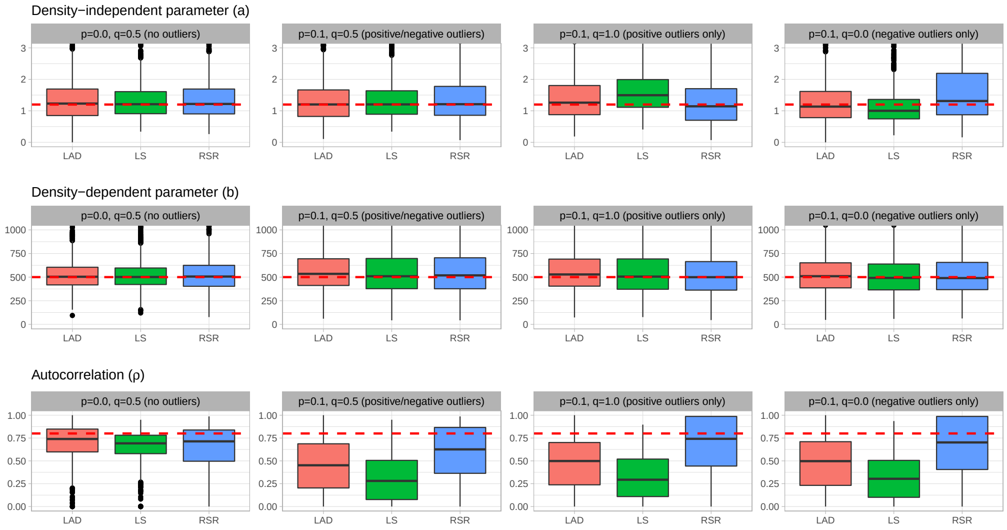

Figure A14: Parameter estimates of the density-independent parameter (a), density-dependent parameter (b), and autocorrelation (ρ) for the simulation using the HS SR function with autocorrelation (true $\rho = 0.8$) in the residuals (S2: $p = 0.1$).

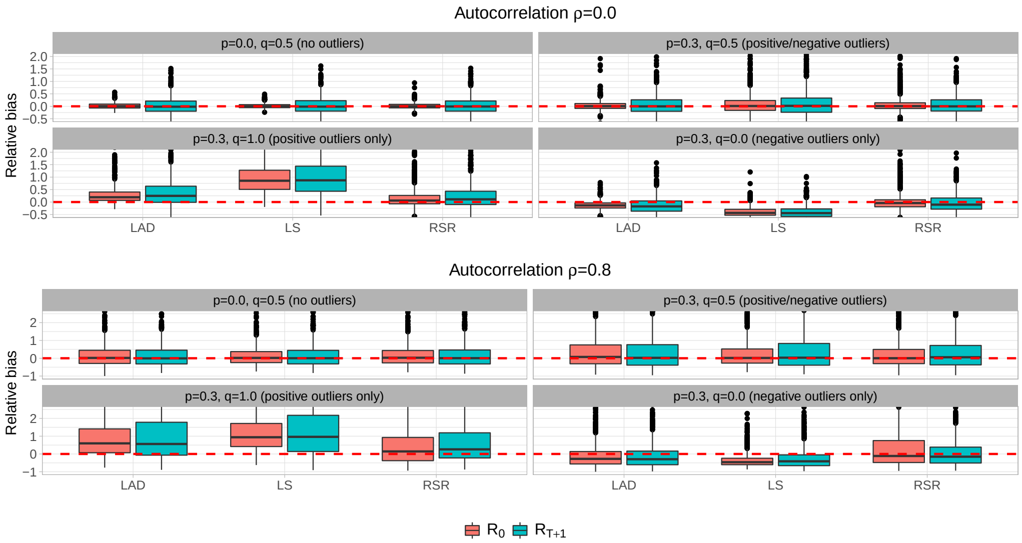

Figure A15: Relative bias of R_0 and R_{T+1} for the simulation using the HS SR function with/without autocorrelation and outliers in the residuals (S3: $p = 0.3$).

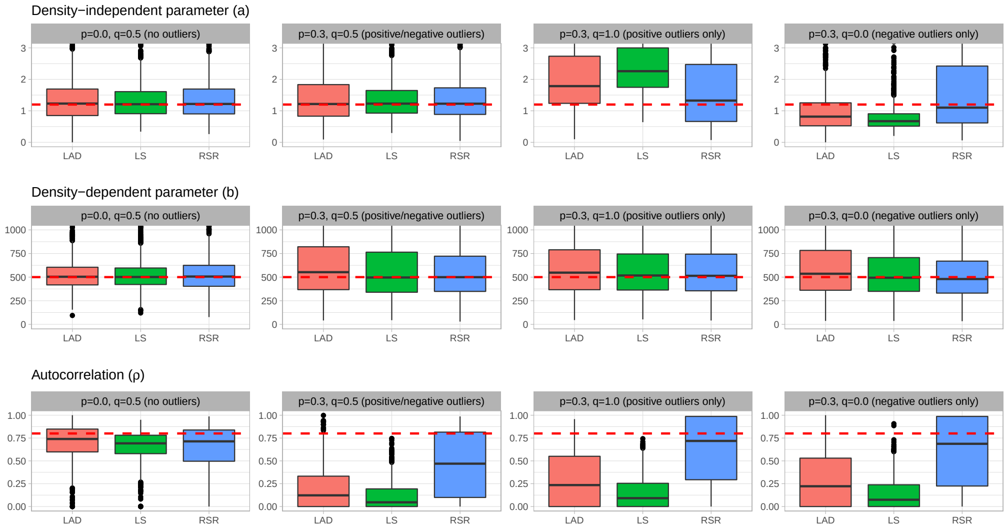

Figure A16: Parameter estimates of the density-independent parameter (a), density-dependent parameter (b), and autocorrelation (ρ) for the simulation using the HS SR function with autocorrelation (true $\rho = 0.8$) in the residuals (S3: $p = 0.3$).

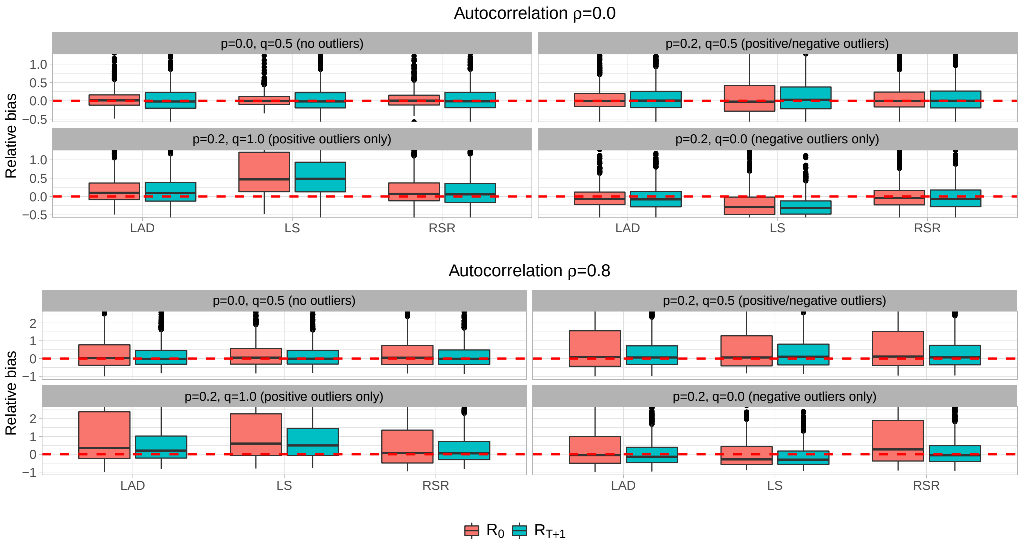

Figure A17: Relative bias of R_0 and R_{T+1} for the simulation using the Beverton-Holt SR function with/without autocorrelation and outliers in the residuals (S4: $f(S_t|\boldsymbol{\theta}) = \log\left(aS_t/(1+S_t/b)\right)$).

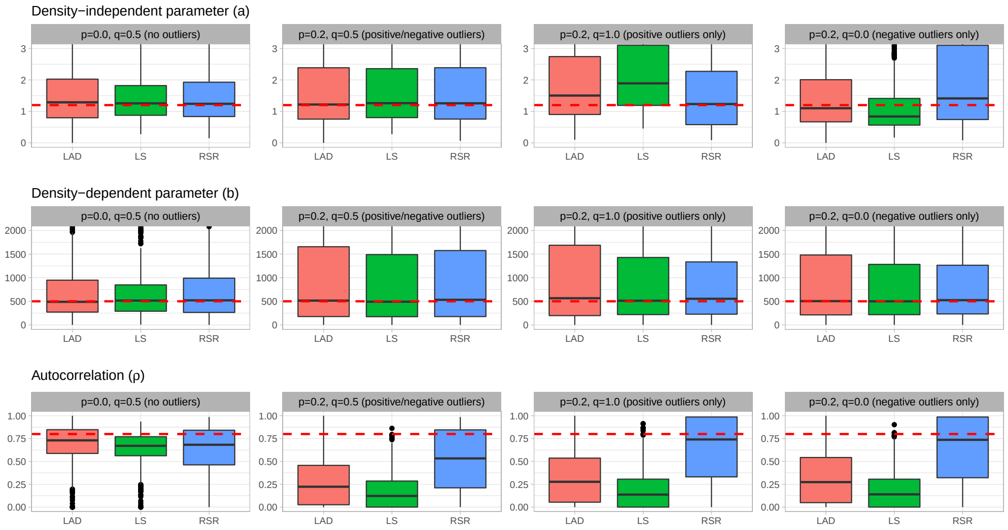

Figure A18: Parameter estimates of the density-independent parameter (a), density-dependent parameter (b), and autocorrelation (ρ) for the simulation using the Beverton-Holt SR function with autocorrelation (true $\rho = 0.8$) in the residuals (S4: $f(S_t|\boldsymbol{\theta}) = \log\left(aS_t/(1+S_t/b)\right)$).

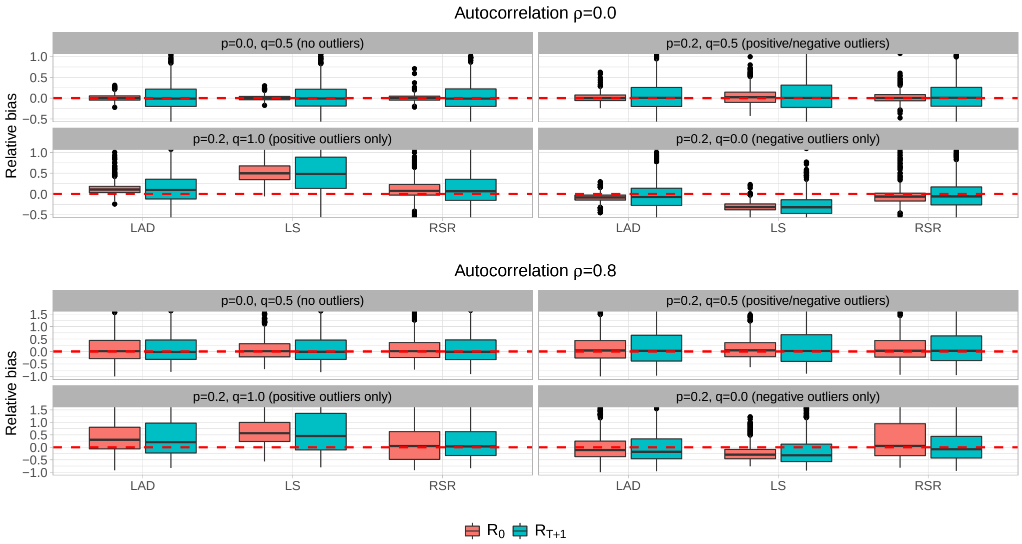

Figure A19: Relative bias of R_0 and R_{T+1} for the simulation using the Ricker SR function with/without autocorrelation and outliers in the residuals (S5: $f(S_t|\boldsymbol{\theta}) = \log(aS_t \exp(-S_t/b))$).

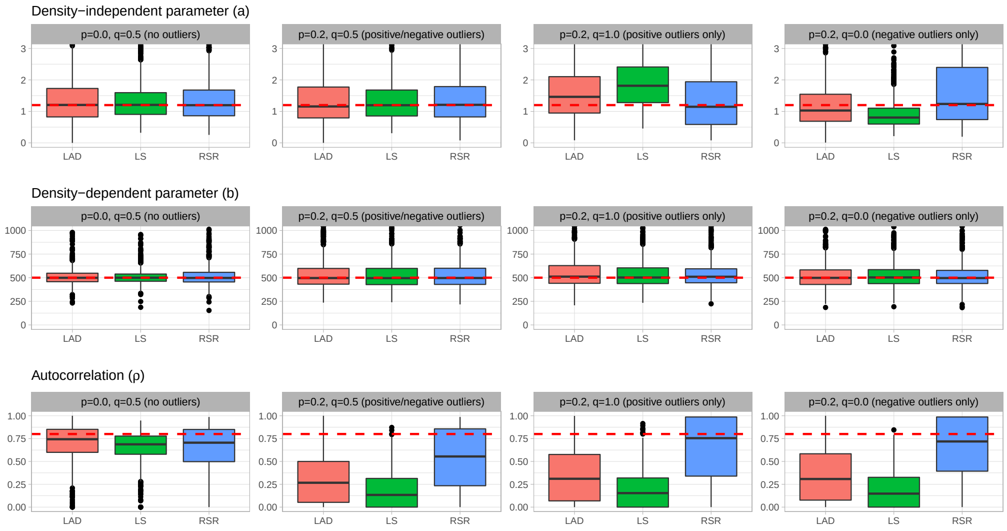

Figure A20: Parameter estimates of the density-independent parameter (a), density-dependent parameter (b), and autocorrelation (ρ) for the simulation using the Ricker SR function with autocorrelation (true $\rho = 0.8$) in the residuals (S5: $f(S_t|\boldsymbol{\theta}) = \log(aS_t \exp(-S_t/b))$).

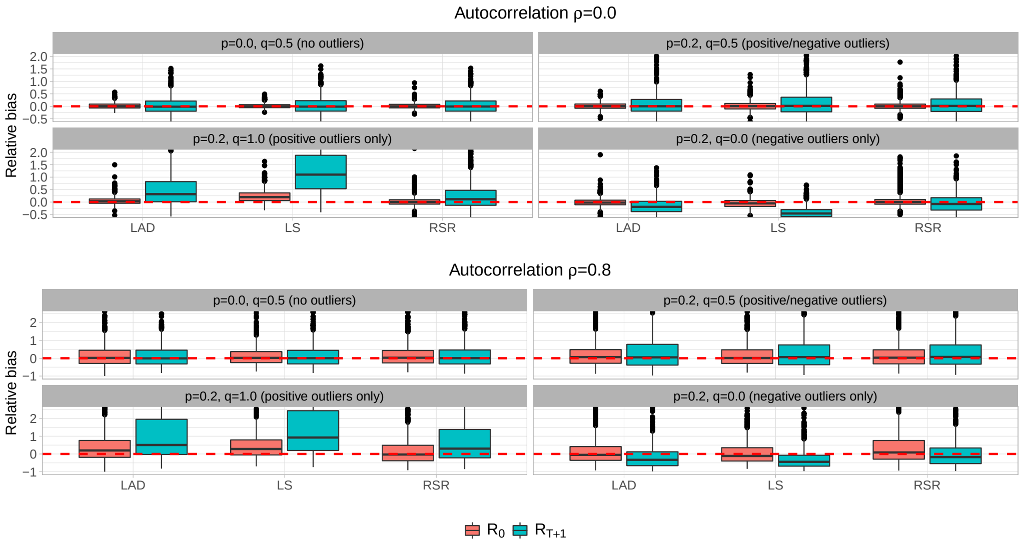

Figure A21: Relative bias of R_0 and R_{T+1} for the simulation using the HS SR function with/without autocorrelation and outliers in the residuals (S6: $\beta = -3.0$ in p_t).

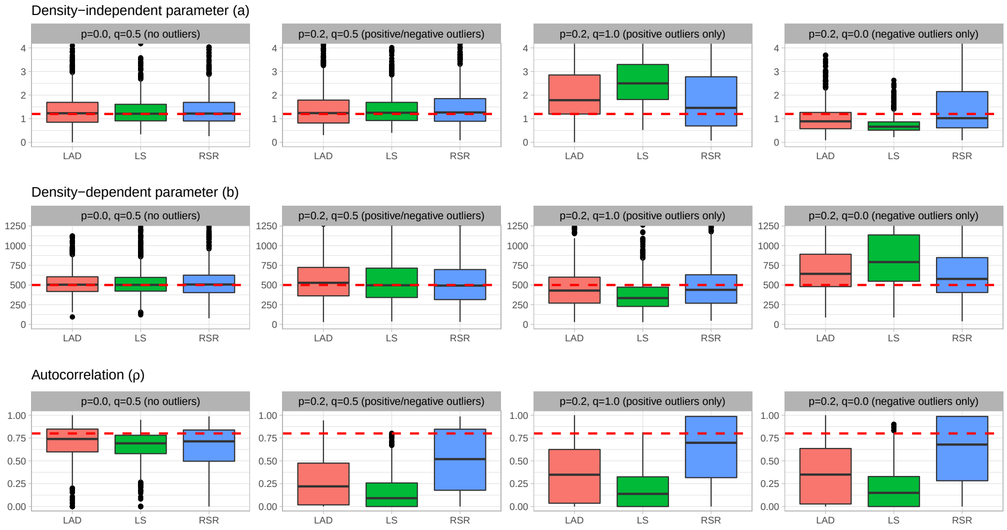

Figure A22: Parameter estimates of the density-independent parameter (a), density-dependent parameter (b), and autocorrelation (ρ) for the simulation using the HS SR function with autocorrelation (true $\rho = 0.8$) in the residuals (S6: $\beta = -3.0$ in p_t).

Appendix H: Estimates of biological reference points for the simulated data

Fishing rate at MSY (F_{msy}) and spawning biomass at MSY (S_{msy}) are calculated from estimated spawner-recrutiment curve parameters a and b assuming the population dynamics model in Appendix D. The relative bias,

$$\frac{\hat{F}_{\text{msy}} - F_{\text{msy}}}{F_{\text{msy}}},$$

and

$$\frac{\hat{S}_{\text{msy}} - S_{\text{msy}}}{S_{\text{msy}}},$$

are evaluated to compare the performances of three time series methods, LS, LAD, and RSR.

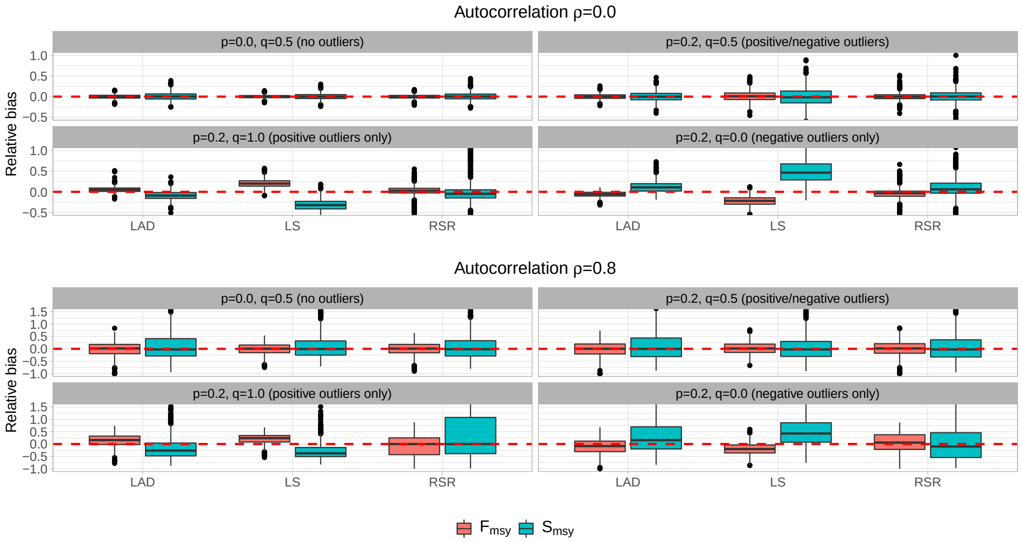

Figure A23: Biological reference points (F_{msy} and S_{msy}) for the simulation scenario S0.

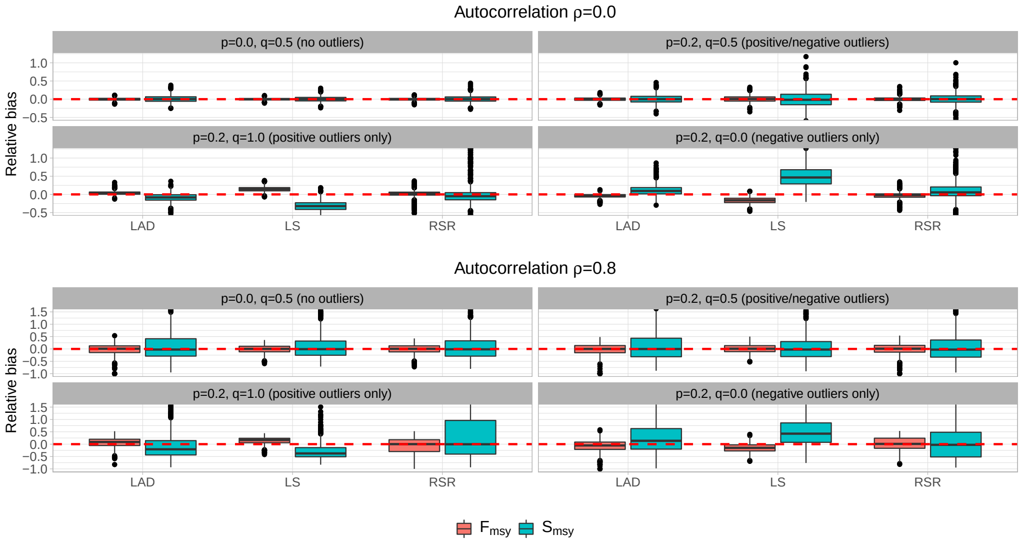

Figure A24: Biological reference points (F_{msy} and S_{msy}) for the simulation scenario S1.

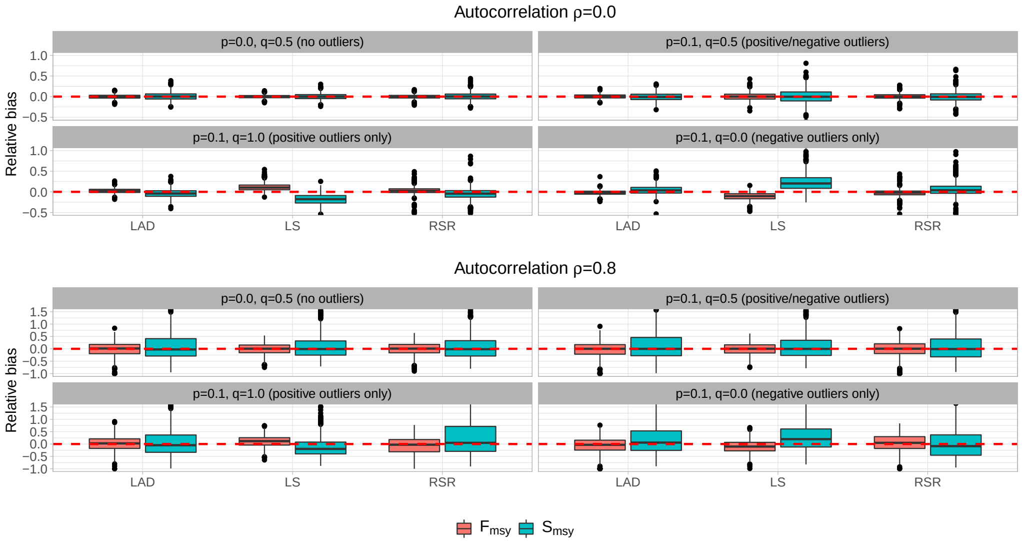

Figure A25: Biological reference points (F_{msy} and S_{msy}) for the simulation scenario S2.

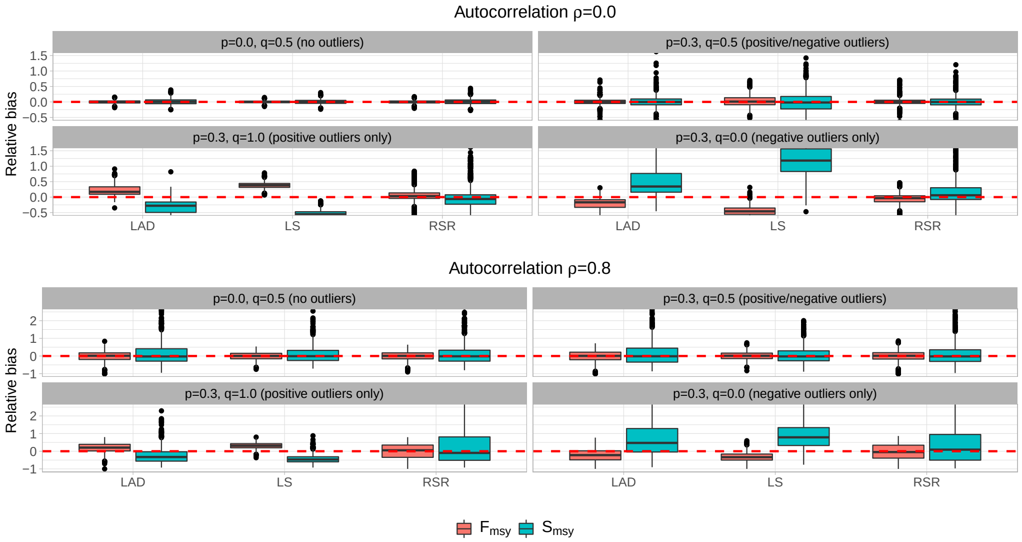

Figure A26: Biological reference points (F_{msy} and S_{msy}) for the simulation scenario S3.

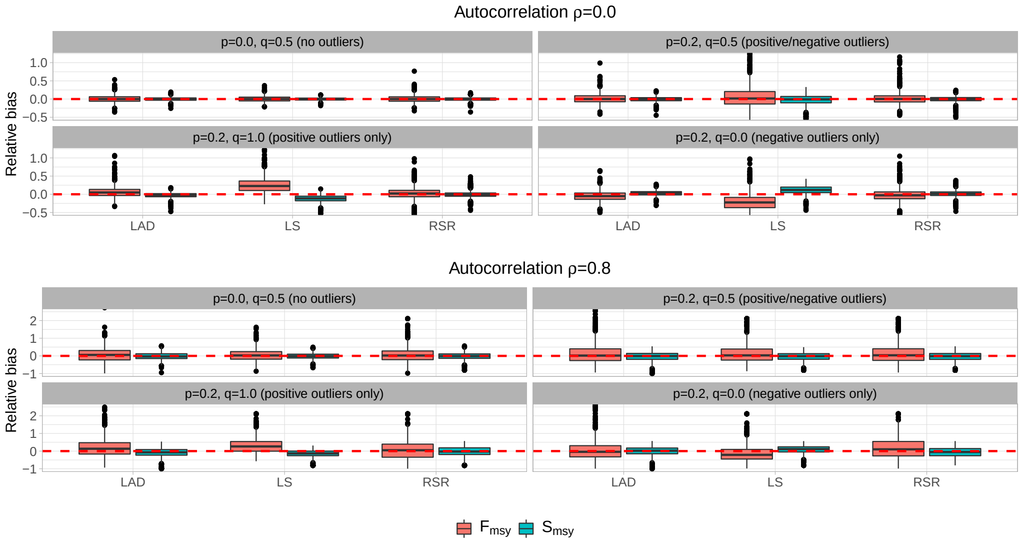

Figure A27: Biological reference points (F_{msy} and S_{msy}) for the simulation scenario S4.

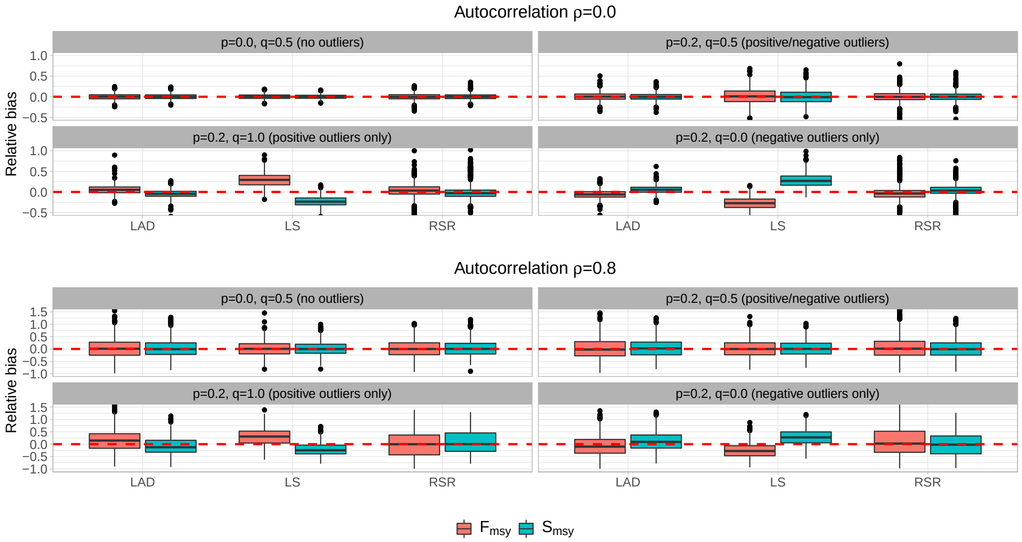

Figure A28: Biological reference points (F_{msy} and S_{msy}) for the simulation scenario S5.

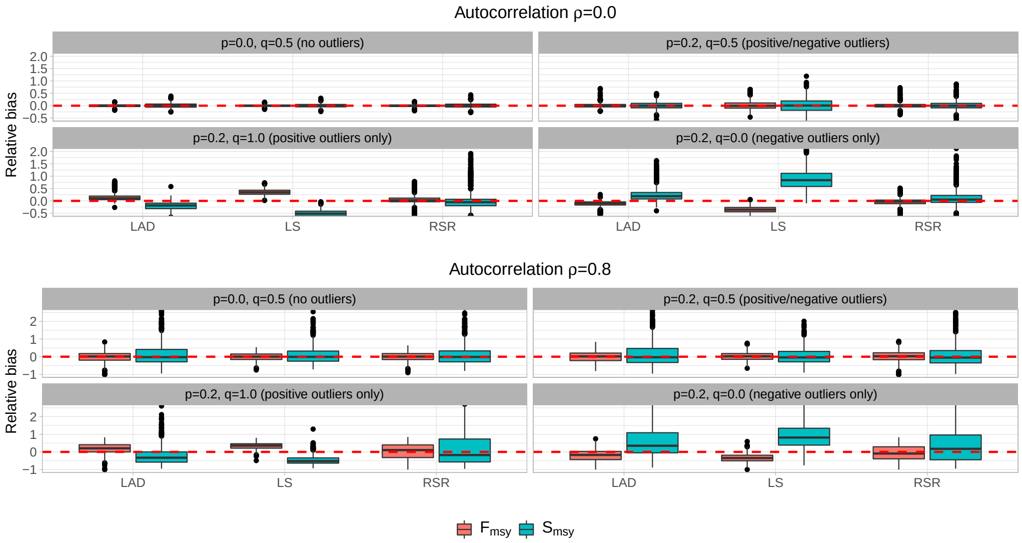

Figure A29: Biological reference points (F_{msy} and S_{msy}) for the simulation scenario S6.
